# Classification of Mammography Images Based on Multifractal Analysis of BIMFs

**DOI:** 10.1155/ijbi/5940783

**Published:** 2025-12-29

**Authors:** Fatima Ghazi, Khalil Ibrahimi, Fouad Ayoub, Aziza Benkuider, Mohamed Zraidi

**Affiliations:** ^1^ Faculty of Sciences LaRI Laboratory, Ibn Tofail University, Kenitra, Morocco, uit.ac.ma; ^2^ Regional Center for Education and Training Professions (RCETP), Laboratory (LAREAMI), Kenitra, Morocco; ^3^ Faculty of Sciences Laboratory of Planet, Animal and Agro-Industry Production, Ibn Tofail University, Kenitra, Morocco, uit.ac.ma; ^4^ Gynecologist-Obstetrician, Coloposcopist, Reference Center for Reproductive Health (RCRHKM), Kenitra, Morocco

**Keywords:** artificial intelligence (AI), bidimensional empirical mode decomposition (BEMD), breast cancer, classification of medical images, computed-assisted diagnosis, Legendre spectrum, multifractal analysis

## Abstract

Breast cancer is a real public health problem. Several women with this disease have died from it. Breast cancer is one of the deadliest cancers. Currently, the only way to combat this scourge is the early detection of breast masses. Mammography is a breast x‐ray that allows images of the inside of the breast to be obtained using x‐rays, thereby detecting possible abnormalities. Computer‐aided diagnosis provides significant support in this direction. This work introduces a new system called MF‐BIMFs for computer‐aided diagnosis that automatically analyzes digital mammograms to discover areas of interest in breast images and offers experts a second opinion. This system was based on the combination of two steps. The first step is the image preprocessing which was based on the bidimensional empirical mode decomposition (BEMD) of breast mammographic images, and their objective is to decompose the image into several BIMF modes and the residual, while the second step is the extraction of features and irregularity properties of the preprocessed images from the multifractal spectrum on each BIMF and the residual and to extract a better representation of each mode and provide details capable of differentiating the two healthy and cancerous states, using these properties as characteristic attributes to evaluate their performance in characterizing two conditions objectively. The rate of this classification is given by SVM. The experimental results indicate that the BIMF_1_ mode provided the best classification rate, approximately 97.32%. The interest of this new approach was applied to real mammographic image data from the Reference Center for Reproductive Health of Kenitra, Morocco (RCRHKM), which contains normal and pathological mammographic images.

## 1. Introduction

The term “cancer” is an umbrella term for a group of diseases that affect the body at the cellular level. These abnormal cells can clump together to form a mass of tissue called a tumor. If the breast is the primary site of a tumor or cancer, it is called “breast cancer.” According to the latest statistics from the World Health Organization, breast cancer is the most common cancer among women, in 157 out of 185 countries, resulting in 670,000 deaths worldwide in 2022 [1]. The widespread implications of breast cancer, including its various financial, physical, emotional, and societal challenges, extend beyond individuals to children, families, communities, healthcare systems, and survivors facing ongoing health problems and the risk of recurrence [2–4]. To further our understanding of breast cancer disparities and their implications, particular emphasis has been placed on highlighting global trends using existing datasets, such as the Global Cancer Observatory (GLOBOCAN) and the Global Burden of Disease [5–12]. Results have consistently highlighted persistent disparities, with many groups historically and systematically overlooked. Although some cases of breast cancer are inevitable, these disparities also result from various modifiable and preventable factors, such as limited access to medical care, socioeconomic status, educational attainment, cultural norms, and individual decisions. However, addressing some of these issues, while theoretically feasible, can be challenging, particularly given the resources required in terms of time, expertise, and funding. Furthermore, in regions lacking appropriate infrastructure and measures, mammography screening for the general population may not be feasible, despite strong recommendations from the WHO and developed countries for annual or biennial screening for people aged 40 years and older [13–16]. Data on breast cancer epidemiology, treatment regimens, and long‐term outcomes allow for the personalization of treatments, identification of treatment gaps, and evaluation of the effectiveness of interventions [17]. In recent years, numerous studies, inspired by the human visual system, have demonstrated the value of digital images in several fields, including medical images in various forms, such as mammography, the main tool for screening and diagnosing breast cancer [18]. There is a wide variety of intraoperative mammography techniques for margin analysis. Artificial intelligence (AI) has the potential to reduce variations in the interpretation of mammograms and optimize diagnostic accuracy [19]. Techniques dedicated to mammographic image processing, as well as computer‐aided diagnosis (CAD) systems, are being developed for this purpose [20]. As computer‐aided image analysis (CAD) is a crucial field, the main means by which a human observer can decipher a visual message are classifications and textures, enabling the description, characterization, segmentation, and analysis of images. A set of properties must be extracted and expressed as parameters. Due to the extreme irregularity of textures, interpreting information in a medical setting is complex. In this context, it is now necessary to propose tools that allow the desired information to be located or processed quickly. There are several methods for characterizing texture that are proposed in the literature, including structural models, statistics, and frequency [21, 22]. For this reason, we have relied on the modeled methods; it is the fractal dimension that allows a global classification between healthy and diseased images [23], but we are not able to characterize the dimension of an object by itself when it presents a point regularity that varies significantly from one point to another; therefore, a strategy based on local analysis must be used; in this regard, multifractal analysis can result in effective image segmentation [24]. The latter can be used to measure the variation of intensity in mammographic images in terms of classifications, descriptions, characterizations, and segmentations. The extremely broad field of multifractal analysis was founded in the 1980s to interpret image signal measurements. It is particularly useful for dealing with the singularities of a medical image and applies to the fields of signal processing and image processing. It notably presents the idea of scale invariance, or the relationship that exists between a measurement and its scale [25]. In general, images contain a large amount of data. To avoid excessively long processing times, we therefore seek to apply local operators, which use, for preprocessing the images and the calculation, only a limited number of pixels located near the image. Therefore, it is important to add the preprocessing step, where the preprocessing algorithms improve the quality of the images.

Huang et al. [26] first suggested a technique called empirical mode decomposition (EMD) for one‐dimensional signals, and 2 years later, it was explicitly extended to image processing (bidimensional empirical mode decomposition [BEMD]) [27], from which it can be extracted into a redundant collection of signals and a residual [28]. It consists of decomposing the image into different modes called BIMFs (bidimensional intrinsic mode functions) [29], and it is possible to recover the original image without any data loss or falsification by integrating all the BIMFs with the residual. This technique has been successfully applied to real data, demonstrating its effectiveness in fields such as oceanography and climate studies [30], image denoising [31], image compression [32], image feature extraction [33], and image texture classification [34], but we are interested in exploring its application in the field of medical imaging.

There are several state‐of‐the‐art research works carried out in the biomedical field related to cancer classification, feature extraction, and preprocessing. Among these works, we find that texture features based on Gabor wavelets and the contourlet transform are the most widely used to extract texture features at different orientations. Texture features based on Gabor filters were proposed by Khan et al. [35] for classification; support vector machine (SVM) was implemented, and an average accuracy of 93.95% was obtained. In the method presented by Liu and Tang [36], geometric and textural features are incorporated into the SVM classifier for mammography mass classification; an accuracy of 94% was achieved with this method. Also, the results of calculating gradient and texture features based on the gradient co‐occurrence matrix (GCM) of mammographic masses obtained a score of 74.4% [37], while the use of texture features for this same categorization via a decision tree gave a result of 93.6% [38], and using the SVM family to classify mammographic masses by contourlet achieved an accuracy rate of 96.6% [39].

For multifractal measurements with the opt‐mBm, ref‐mBm, DBC, blanket, variance, multifractal measurement, and opt‐mBmVariance methods, the respective values for the area under the ROC curve (AUROC) are 0.919, 0.707, 0.494, 0.747, 0.843, 0.561, and 0.945 [40]; a spatial multifractal spectral distribution approach for the classification of breast ultrasound images has achieved an accuracy rate of 94.8% [41], and with the use of BEMD for computer‐aided diagnosis of breast cancer has achieved an accuracy rate of 95% [42].

With preprocessing and combination with other models, followed by feature extraction, Eltoukhy and Faye [43] proposed an Otsu method for segmentation with a reduction of false positives (FPs) in which features are extracted by both methods: curvelet and wavelet, with an accuracy of 96.66% with an SVM classifier and 93.37% with KNN. Jebasonia and Dheeba [44] used the same preprocessing and segmentation methods as us, but for the feature extraction method, they used the texture energy measure (LTEM) with an accuracy of 95.89% with SVM. Khan et al. [45] used the rotation‐invariant LBP method combined with the uniform model for feature extraction and the SVM method for classification, with a 74% retention rate. dos Santos Teixeira [46] used grayscale reduction. Features were calculated by a co‐occurrence matrix and SVM as a classifier with a rate of 85%. With preprocessing, two techniques by BMED and MBMD are used, followed by feature extraction for the classification of masses in benign or malignant mammograms with an accuracy of 90% and 92.59%, respectively [47]. In a study on detection using fractal dimension measures of BEMD in digital mammography [48], an accuracy of 95% was obtained. The use of texture features combined with a Markov random field (MRF) model [49] had an accuracy of 94%, and a cancer diagnosis by CSVM had an accuracy of 84% [50]. The result of the segmentation reaching a rate of 83.62% accuracy is obtained, thanks to a lightweight U‐Net network built on a CNN [51]; a breast cancer detection system based on a CNN‐CBR architecture for the classification of mammograms has achieved an accuracy of 86.71% [52]; with the use of multi‐instance learning of deep convolutional neural networks for the classification of whole slides in breast histopathology, an accuracy of 89.52% is achieved [53], and with the use of convolutional neural networks and transfer learning to classify breast cancer on histopathological images using a graphics processing unit, a rate of 84% has been obtained [54]. The objective of this article is to improve classification performance. The goal is to develop a diagnostic support system for the detection and classification of masses in mammographic images, to propose tools to quickly locate or process the necessary or desired information, and to help radiologists and physicians improve the accuracy of mammographic image interpretation. The integration of AI then enables decision‐making and classification of these images.

The originality of this article lies in the combination of a BMED preprocessing phase called EMBD. This decomposition allows for signal and image analysis. It consists of decomposing the signal and image based on functions called intrinsic mode functions (IMFs) and two‐dimensional intrinsic multimodal functions (BIMF_*k*_), respectively. The unique feature of this method lies in the fact that the basis for the decomposition is not given a priori but rather constructed from the properties of the signal (image) itself. Next, applications of two‐dimensional multimodal empirical decomposition (BMED) in image processing were presented. This approach is motivated by the search for a good interpretation in order to extract characteristic information from the image under study and, secondly, by an attribute extraction phase. This last phase is based on the measurement of a multifractal index, the Hölder index, which must be calculated and indicates the overall accuracy of the BIMFs to be created from the residual, the original image without preprocessing, and the reconstructed image, where multifractal analysis proves more suitable for discriminating preprocessed mammographic images. Finally, a classification is performed using these attributes to objectively assess the ability to characterize two conditions: healthy and cancerous. The quantitative parameter of the characterization will be the classification rate, calculated by SVM. Our approach was tested and validated on mammographic images from the Reference Center for Reproductive Health of Kenitra, Morocco (RCRHKM), which contains mammographic images of real breasts of normal and cancerous cases.

The organization of this paper is as follows: In the second section, we provide the theoretical framework, which includes a summary of multifractal analysis and a theoretical study of BEMD. Section 3 gives the SVM classifier; the general architecture of the proposed approach is presented in Section 4; the results, along with their interpretations, are in Section 5; and the conclusion and outlook are given in Section 6.

## 2. Theoretical Frameworks

### 2.1. Multifractal Spectrum

#### 2.1.1. Multifractal Analysis of Images

Multifractal image analysis involves defining measurements from gray levels, calculating the spectrum, and processing the points based on the resulting local and global information [40]. The local description was obtained via the Hölder exponent. The overall description consists of measuring the size of the subsets thus obtained, that is to say, studying the sets *E* *α*. Thus, to obtain the multifractal spectrum of the distribution of singularities of a signal, there is continuity and differentiability at a point. Texture analysis using the Hölder exponents is another tool that has a stronger theoretical basis and logical content [55]. In image processing, this is the case when we seek to detect contours in an image; the greater the value of our exponent, the more regular the image.

#### 2.1.2. Difficulty of Fractal Analysis

The fractal dimension is not precise, whatever the method used, because the image is assumed to be a surface in a three‐dimensional Euclidean space, such that (*x*, *y*) represents the pixel and *z* represents the gray level. This approach has no theoretical support because we cannot consider light intensity as a geometric dimension. After all, it reflects the physical properties of the pixels. A solely fractal approach does not take into account the different local fractal behaviors that embody the different irregularities that exist in the image. A multifractal analysis was developed to correct errors by considering the light intensity associated with each pixel as a measurement based on a parameter *α* (characterizing the “singular” behavior of gray levels). So the couple (*α*, *f* (*α*)) allows us to characterize the texture of an image.

#### 2.1.3. The Singularity of a Signal

The Hölder exponent is a measure of singularity. A measure of the measurement’s local scale invariance defined on the image is called a [56] point or local. The local fractal dimension is measured by singularity, which tells us about the regularity of the image around a point (*x*, *y*).

At the point (*x*, *y*), the local Hölder exponent *α*(*x*, *y*) provides the singularity (Equation 1).

(1) αx,y=limdt⟶0logμBx,y,dtlogdt,

where *B*((*x*, *y*), *d* *t*) is the ball with center (*x*, *y*) and radius *d* *t*, the estimation of *α* is done by linear regression of all the coordinates, and *μ* is the measurement that is a function of gray level.

#### 2.1.4. The Multifractal Spectrum

The Hölder exponent of a local approach to images is at best. This only provides valuable information on the local irregularity of the image.

A characterization of points of the same singularity and the distinction in an image of the different local behaviors gives us a better description of the images. Then, we consider that each value **α** of the Hölder exponent defines a fractal set **E**(**α**) (Equation 2). It has a fractal dimension that we will compute and whose support consists of various fractal sets, in fact, either of the following:

(2) Eα=x,yαx,y=α with x=∪αEα.

The subsets of points with the same scaling behavior as characterized by *α* are represented by *E*(*α*). In certain situations, obtaining the information offered by the Hölder function *α* (*x*, *y*) is either challenging or unfeasible. It is preferable to describe from a geometric or statistical point of view the distribution of the Hölder exponents of the image: This approach is called multifractal analysis. Indeed, three types of multifractal spectra are usually defined: the Hausdorff spectrum, the spectrum of large deviations, and the Legendre spectrum.

#### 2.1.5. The Hausdorff Spectrum

For each value *α* of the Hölder exponent, a fractal set *E*(*α*) is defined. The Hausdorff fractal dimension is defined by Equation (3).

(3) fhα=dimHEα,

where dim **is** the Hausdorff dimension. The couple (*α*, *f*_*h*_(*α*)) provides local (via *α*) and global (via *f*_*h*_(*α*)) information. The Hausdorff spectrum (*α*, *f*_*h*_(*α*)) provides a geometric characterization of the distribution of singularities of the image signal. The range of significant deviations and the spectrum of large deviations statistically characterize the single (Equation 4).

(4) αnk=−logμInknlog2.

We want to evaluate the probability of a given exponent when we choose an interval *I* at random in [0,1] or the evolution of this probability when *n* tends towards infinity. The double characterization (*α* and the likelihood of finding *α*) makes it possible to give variations of the image signal.

#### 2.1.6. Legendre’s Multifractal Spectrum

The image is considered a measurement distributed on a compact medium. Thus, we further define *μ* as a Borel probability measure on the compact [0, 1] × [0, 1] and *v*_*n*_ by an ascending series of positive integers. We further define the windows *I*, *j*, and *n* according to the following formula (Equation 5):

(5) Ii,j,n=ivn,i+1vn×jvn,j+1vn.

If we consider the quantity (Equation 6),

(6) τnq=−1logvnlog∑i,jμIi,j,nq.

We say that the measure **μ** has multifractal behavior if Equation (7) is as follows:

(7) limn⟶∞τnq=τq.

Multifractals are different from fractals in two significant areas:

– They are based on sets of measurements and not on a fractal dimension, as in the case of fractals.
– They provide a spectrum of fractal dimensions.

Therefore, it is important to find the distribution of singularities of a multifractal **f** to analyze its properties.

The spectrum of **f** is given by the following: Let **S** be the set of every actual place where the point Lipschitz regularity of **f** is worth **α**. The spectrum of singularities **D**(**α**) is the set of **α** for which **S** is nonempty. This spectrum of singularities was defined by Martinez et al. [57]; however, it is impossible to calculate the Lipschitz regularity of a multifractal because its singularities are not isolated. We therefore use a partition function to calculate the spectrum. For the implementation, we reused the box method. First of all, we had to introduce a measurement, denoted by **μ**.

To do this, we calculated the probability of discovering a pixel in a box of fixed size **r** (Equation 8):

(8) Pir=LirLTr,

where *L*_*i*_ is the number of pixels in an *r*‐sized box and *L*_*T*_ is the total number of pixels in all boxes.

Thus, we could write the normalized measure (Equation 9) as follows:

(9) μiq,r=Pirq∑i=1NPirq.

Furthermore, the spectrum of generalized dimensions for a specific set was (Equation 10) as follows:

(10)  Dq,r=1q−1limr⟶0log∑i=1NPirqlogr.

Consequently, the subset *f*(*q*, *r*)’s fractal dimensions are found in Equation (11).

(11) fq,r=limr⟶0∑i=1Nμiq,r×logμiq,rlogr

indexed by the exponent **α**(**q**, **r**) (Equation 12):

(12) αq,r=limr⟶0∑i=1Nμiq,r×logPiq,rlogr.

The determined *α* exponents are called the singularities of the set studied.

### 2.2. Multimodal Empirical Decomposition of One‐Dimensional Signals

This section is devoted to the presentation of empirical modal decomposition in its original form [27] by highlighting the key concepts of the method.

#### 2.2.1. Principle

The EMD decomposition can be seen as the recursive application of an elementary decomposition operation, making it possible to extract from any oscillating signal the component that oscillates faster and that which oscillates slower, from which we can again extract the component that oscillates faster. We thus obtain the recursive principle of the EMD, which can be defined as follows:

EMD signal=fast oscillation+EMD slow oscillation.

We can formulate the previous principle by (Equation 13) the following:

(13) xt=∑k=1nxkt+rt,n∈N,

where *x*(*t*) is the original signal, *x*_*k*_(*t*) is the *k*th oscillation called IMF (or mode), and *r*(*t*) is the remainder of the signal *x*(*t*) after extracting all the oscillating components; it is often called “residual,” and *n* is the number of components (IMFs).

The decomposition stops when there is no longer any oscillating signal to be decomposed. Thus, a signal can be spatially characterized as a series of contributions from both slow (low frequencies) and fast (high frequencies) oscillations so that each component locally contains oscillations of lower frequency than those extracted previously.

To illustrate the decomposition, we consider the simple example of a signal *x*(*t*) made up of two contributions: a fast oscillation component and another slow one (Equation 14).

(14) xt⏟signal=bt⏟slow oscillation +ht⏟fast oscillation .

**Figure 1:**
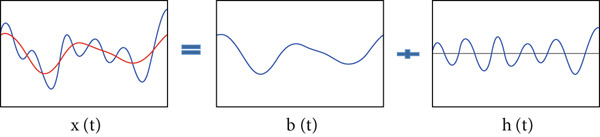
Principle of EMD: slow oscillation + fast oscillation.

**Figure 2:**
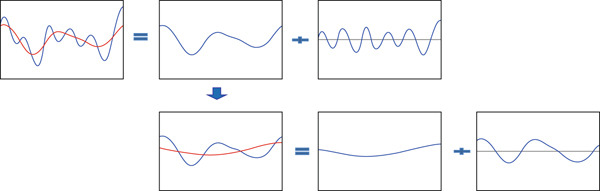
Principle of EMD: decomposition of the signal *x*(*t*).

Thus, rebuilding the signal *x*(*t*) is carried out by summing the two curves *h*(*t*) and *b*(*t*) (Figure 1). To obtain a decomposition of the signal into several components, it is sufficient to repeat the process on the slow oscillation to write the signal as a finite combination of oscillations (Figure 2).

The originality of this method, compared to a wavelet transformation, for example, is that it is not based on a choice of frequency filters fixed a priori but is directly driven by the data (data‐driven). This is why the name intrinsic modal function (IMF) was chosen.

Let IMF be a function:

f:R⟶Rt⟶ft

which satisfied the following properties:

i- The local mean of *f* is zero. This condition is to ensure that the instantaneous frequency (IF) does not include unwanted signal fluctuations induced by asymmetric waveforms.
ii- The number of maxima and zeros differs at most by 1 (which means that between two consecutive extrema of *f*, there is necessarily a zero crossing, then guarantees the narrowband criterion [58].
iii- It follows a modulation law in amplitude and frequency (oscillating behavior) that is naturally of the single‐component type. It is sufficient for the notion of FI to have a physical meaning. Let *m*_*k*_ be the bandwidth of a signal of order *k*. The number of zero crossings per unit time of the signal is then given by the following (Equation 15):

(15) N0=1πm2m0.

The number of signal extrema is calculated by the following (Equation 16):

(16) N1=1πm4m2.

The signal bandwidth, denoted by *L*, is finally given by the following (Equation 17):

(17) L=πN12−N02=m4m0−m22m2m0.

#### 2.2.2. EMD for Images

The extension of the EMD to the two‐dimensional case (BEMD) is introduced by Nunes. The concept of EMD remains the same for the 2D case. This decomposition makes it possible to extract structures at different scales and spatial frequencies, including amplitude and frequency modulations. EMD, in its 2D version, has made it possible to create novel strategies for the multiscale and processing analysis of images such as content‐based image retrieval [59] and 3D medical images [60].

#### 2.2.3. Algorithm

The empirical modal decomposition in 2D is carried out as follows.

Consider a 2D signal (image).

The EBMD algorithm is described as follows:

1. *I*_*r*_ = *I* (*I* is the initial image, and *I*_*r*_ is the residual image).
2. Construct the average envelope of *I*_*r*_.

Let us calculate the maximum and minimum values by interpolating the upper and lower envelopes of *I*_*r*_. Calculate the mean envelope value between the two envelopes.

3. Take the leftover image and subtract the average envelope from it.
4. Repeat from 1 to 4 until the residual image is a BIMF.

The procedure is refined with a loop iterating Steps 1 through 4, called 2D sifting. We iterate until the local average is zero, thus obtaining a BIMF. Thus, the reconstruction of image *I* is carried out by summing all the components ${\left(\text{BIM}{\text{F}}_{k}\right)}_{k=11\cdots .n-}$ and the residual *I*_*r*_ (Equation 18).

(18) I=∑k=1nBIMFk+Ir,n∈Ν,

where BIMF_*k*_ is the *k*th oscillation, *I*_*r*_ is the decomposition residue, and *n* is the number of BIMFs.

#### 2.2.4. The Criterion for Stopping Decomposition

When there are no more oscillations to extract, the decomposition often finishes when the number of extrema is fewer than 2 [27]. There are situations in which we can have a large number of BIMFs without this requirement being met. So we must a priori set a maximum number of BIMFs to extract. The choice of the maximum number is linked to the specifics of the applications. In denoising, for instance, images, we only must come first BIMF [61].

**Algorithm 1: The sieving process algorithm for a given signal *x*(*t*).**
 • i=0, di= x(t),
 • mi = mean (EnvMin(di); EnvMax(dj))
 • di+1 = di‐ mi
as long as d_i+1_ is not a BIMF, do i←i +1
where i is the number of iterations determined according to a certain criterion to obtain the kéme MFI

The sieving is repeated several times (*i* times) until an IMF (BIMF) is obtained. Therefore, the criterion for stopping the sieving process is based on the properties of an IMF (BIMF). Also, the quality of the extraction of a BIMF depends on the quality of the BIMF’s previous ones. Consequently, the choice of the criterion for stopping the sieving process becomes very important. In this context, several proposals have been put forward:

• Assume the existence of the convergence of the sieving process.
• Perform a certain number of iterations without any validation testing on the IMF (BIMF) extracted (approach not recommended).
• Define a stopping criterion during sieving.

The author [8] proposes a stopping criterion SD(*j*) based on the standard deviation (SD) defined by (Equation 19):

(19) SDj=∑t=0Tdj−1m,n−djm,n2dj−12m,n+ξ,

Where is the *j* *^t^* *h* IMF (BIMF), *d*_*j*−1_(*m*, *n*) and *d*_*j*_(*m*, *n*) are the results of two consecutive sievings, and *ξ* is a (weak) term intended to eliminate possible divisions by zero. We can view this stopping criterion as a measure of the relative error on l^’^IMF_*j*_(BIMF_*j*_). Ideally, we would like this error to be as small as possible.

This stopping criterion is valid when SD(*j*) is below a certain threshold; SD_max_ is predefined as follows (Equation 20):

(20) SDj<SDmax.

In practice, the value is chosen empirically. Typically, Huang et al. suggested taking a value between 0.2 and 0.3. This value gives satisfactory results.

#### 2.2.5. Application Examples

In this section, we will present examples of BEMD applications. The first example illustrates the principle of BEMD decomposition, that is, a multiscale decomposition from high to low frequencies. Empirical modal decomposition in 2D is performed as follows: consider a 2D signal (image).

Let an IMF be a function:

f:R2⟶RX⟶fX

which verifies the following properties:

1. List every extreme in the area of *f*(*X*).
2. Interpolate the minima (resp., the maxima) to construct the envelope lower “EnvMin” (resp., upper “EnvMax”).
3. Determine the average *m*(*X*) = (EnvMin(*X*) + EnvMax(*X*))/2.
4. Take out the specifics *d*(*X*) = *f*(*X*) − *m*(*X*).
5. Iterate over the residue *d*(*X*) (until *m* is zero or *d*(*X*) is a BIMF).

As in the 1D case, the procedure is refined with a loop iterating Steps 1 through 5, called 2D sieving. We therefore iterate over the detail *d*(*X*) until the local mean *m* is zero, thus obtaining a BIMF (2D IMF). The image *f*(*X*) is therefore first decomposed in the main loop as follows:

(21) fX=d1X+r1X.

The first residue *r*_1_(*X*) is then decomposed as follows:

(22) r1X=d2X+r2X.

Finally, *f* is decomposed as follows:

(23) fX=∑k=1NdkX+rX,

where *d*_*k*_ denotes the *K*th BIMF and *r* is the residue.

**Figure 3:**
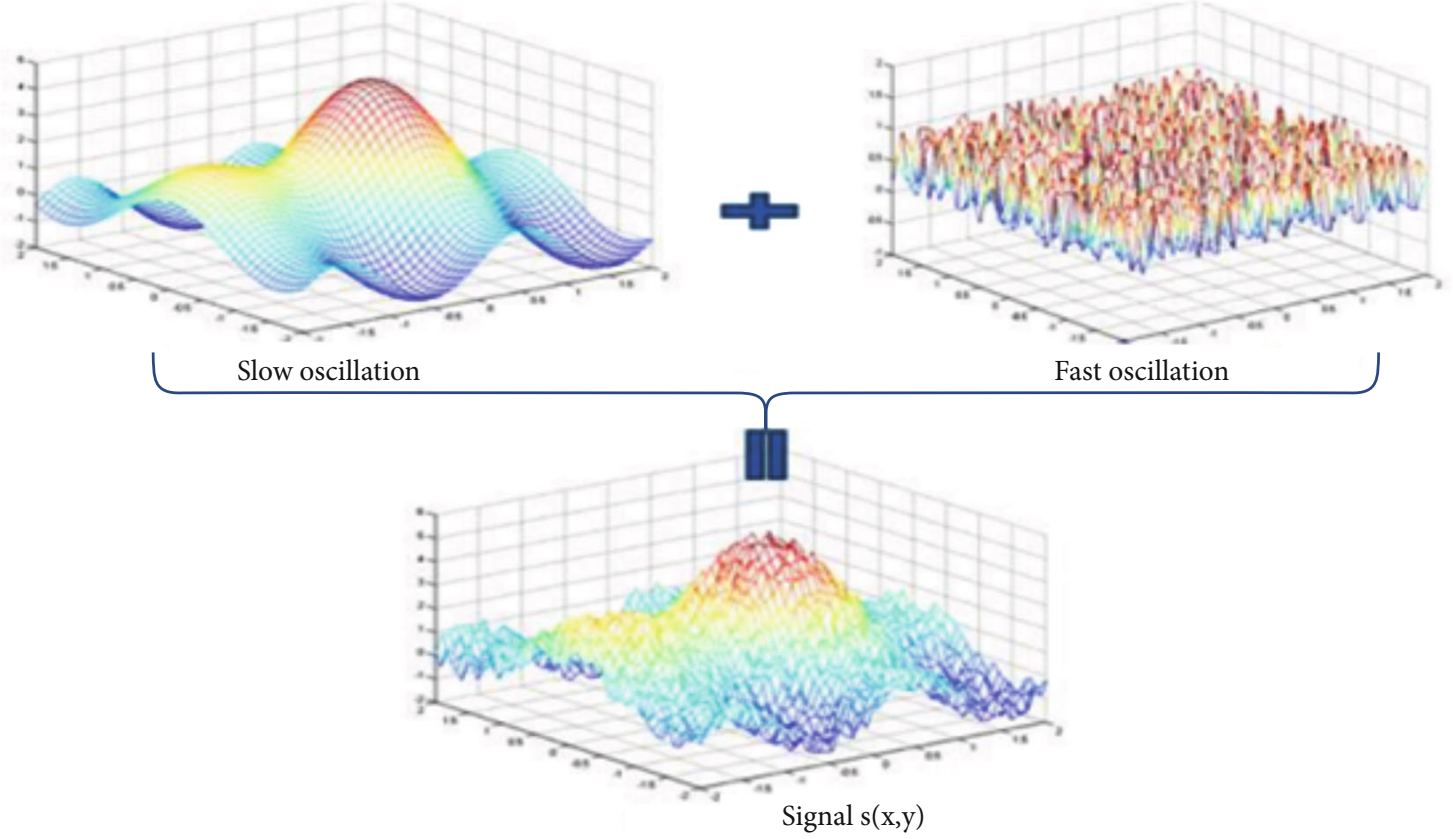
Example for a multiscale decomposition from high frequencies to low‐frequency signal *S*(*x*, *y*): fast oscillation + slow oscillation detail.

Figure 3 illustrates an example of a synthetic 2D signal (Equation 24) composed of a fast oscillation and a slow oscillation.

(24) Sx,y=exp−x2+y2+cos2y+sin3x+bruit_blancx,y.

In an image, a pixel has eight neighbors. We define the neighbors of a pixel in an image by a 4‐connectivity or 8‐connectivity type. The criterion for determining whether the pixel is a local maximum (minimum) is to take each pixel and then compare it to its neighbors, using one of the two connectivity types. The extrema must be strictly greater or smaller than all of their neighbors. The operation of finding extremum points can be very computationally expensive for large images. Hence, the interest in seeking alternative methods, such as morphological reconstruction, is one of the alternative methods used to find extreme points. Geodesic operators (dilation and erosion) are the basis of this technique [62, 63]. For an image *I*, the morphological dilation with the structuring element *B* is defined as follows (Equation 25):

(25) δBI=I⊕B=supIx−qx∈I,q∈B.

The same applies to the morphological erosion of image *I* with the structuring element *B*. It is defined as follows (Equation 26):

(26) δBI=I Θ B=infIx−qx∈I,q∈B.

The minimum and highest values found locally of the signal *s*(*x*, *y*) are presented in Figure 4a,b, respectively.

Figure 4Stages of signal decomposition for extraction of extrema: (a) “extremum max” and (b) “extremum min” of a 2D signal (morphological reconstruction).

**Figure:**
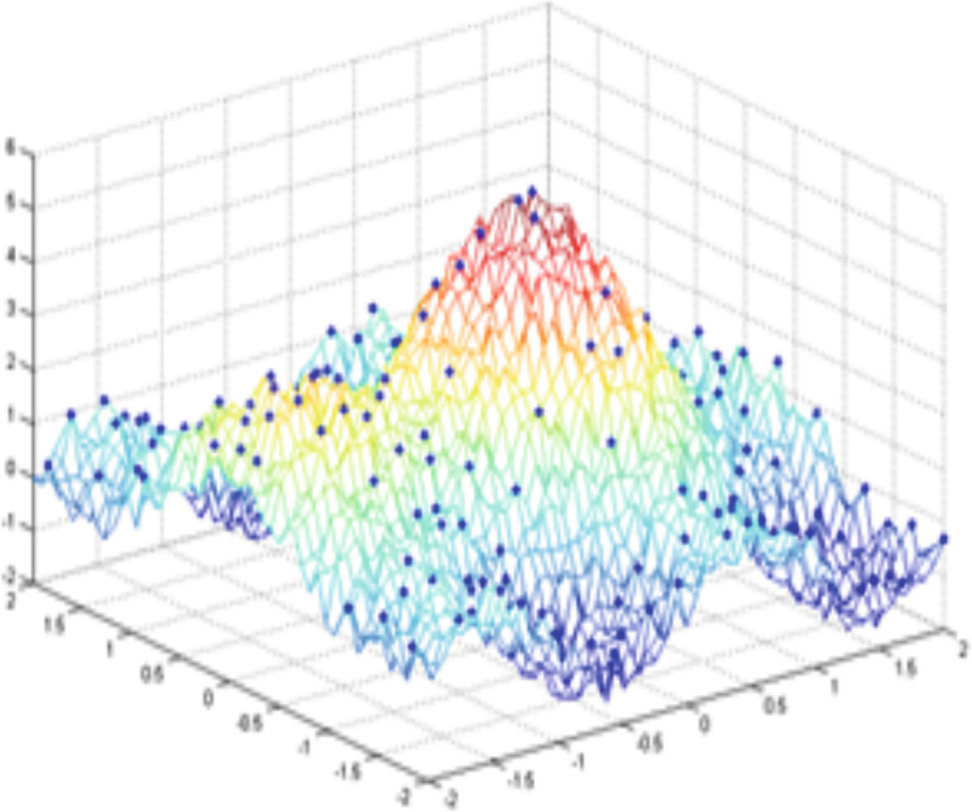
(a)

**Figure:**
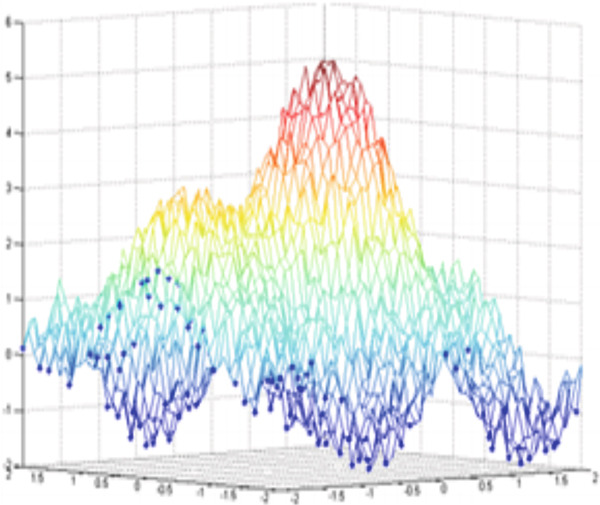
(b)

After the extremum extraction step, we perform interpolation, one of the key steps for estimating and extracting the IMFs (BIMFs) from the extrema. Once the extrema have been extracted, they must be interpolated to obtain the upper and lower envelopes. In the two‐dimensional case, the points to be interpolated are not arranged on a regular grid but are scattered (scattered data), which requires a more sophisticated method adapted to this type of problem. Huang proposed using cubic splines on nonequidistant data for one‐dimensional signals, but its application in the 2D case results in edge discontinuities in the reconstructed image. In general, radial basis functions (RBFs) are used [64].

A RBF is of the form (Equation 27):

(27) ∀x∈Rd;Sx=pmx+∑k=1nλkϕx−xk,with xk∈Rd et λk∈R,

where *p*_*m*_ is a low‐degree polynomial, *x*_*k*_ with 0 ≤ *k* ≤ *n* are the interpolion centers, ‖.‖ is the Euclidean norm in $R^{2}$, *ϕ* is the basis function for functions with an assumed fixed radial basis, and *λ*_*k*_ is the RBF coefficients to be determined.

The equation *S*(*X*) resulting from an RBF interpolation is defined by the coefficients of the polynomial *P* and the weights *λ*_*k*_. Given *f* = (*f*_1_, *f*_2_, ⋯⋯.*f*_*N*_), we look *λ*_*k*_ for the weights for the RBF to check (Equation 28):

(28) sXi=fii=1,..⋯⋯,N,with Xi=xi,yi.

By doing this, we can reduce our problem from one in an infinite‐dimensional space to one in a finite‐dimensional space (the set of coefficients that determine *s*) and to a linear system that can be solved using standard linear algebra techniques. The figure below illustrates the upper, lower, and middle envelopes (Figure 5a,b, respectively).

Figure 5Construction of the envelopes: (a) in red the upper envelope and in blue the lower envelope; (b) the middle envelope.

**Figure:**
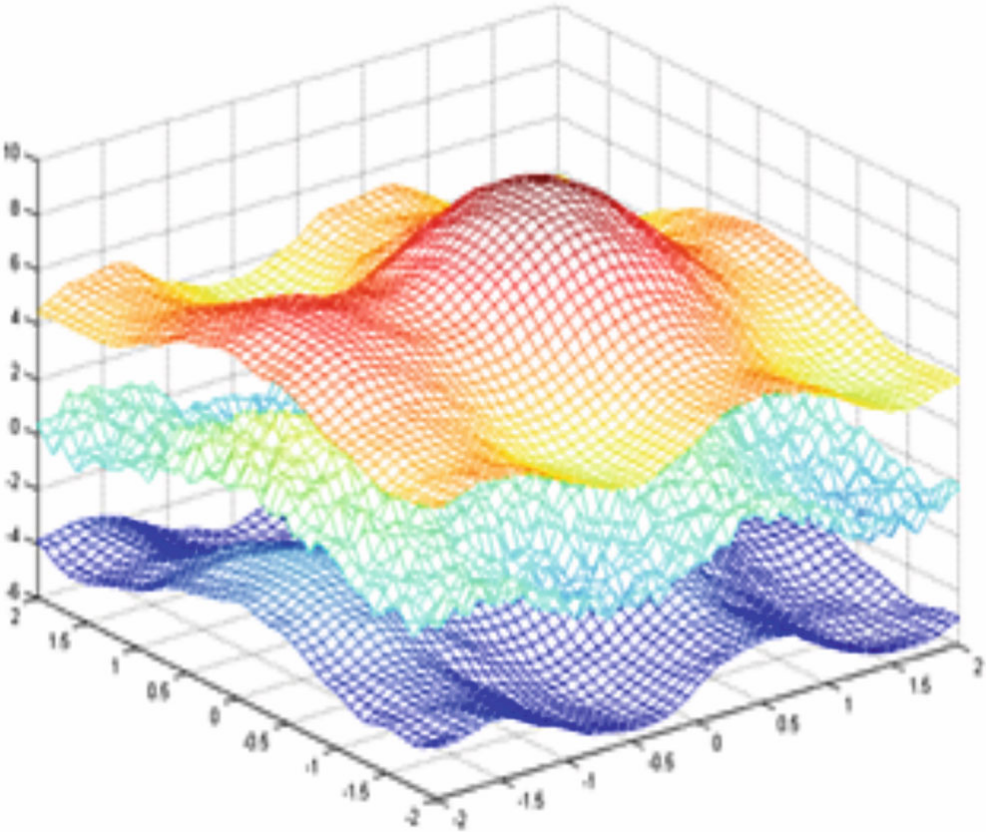
(a)

**Figure:**
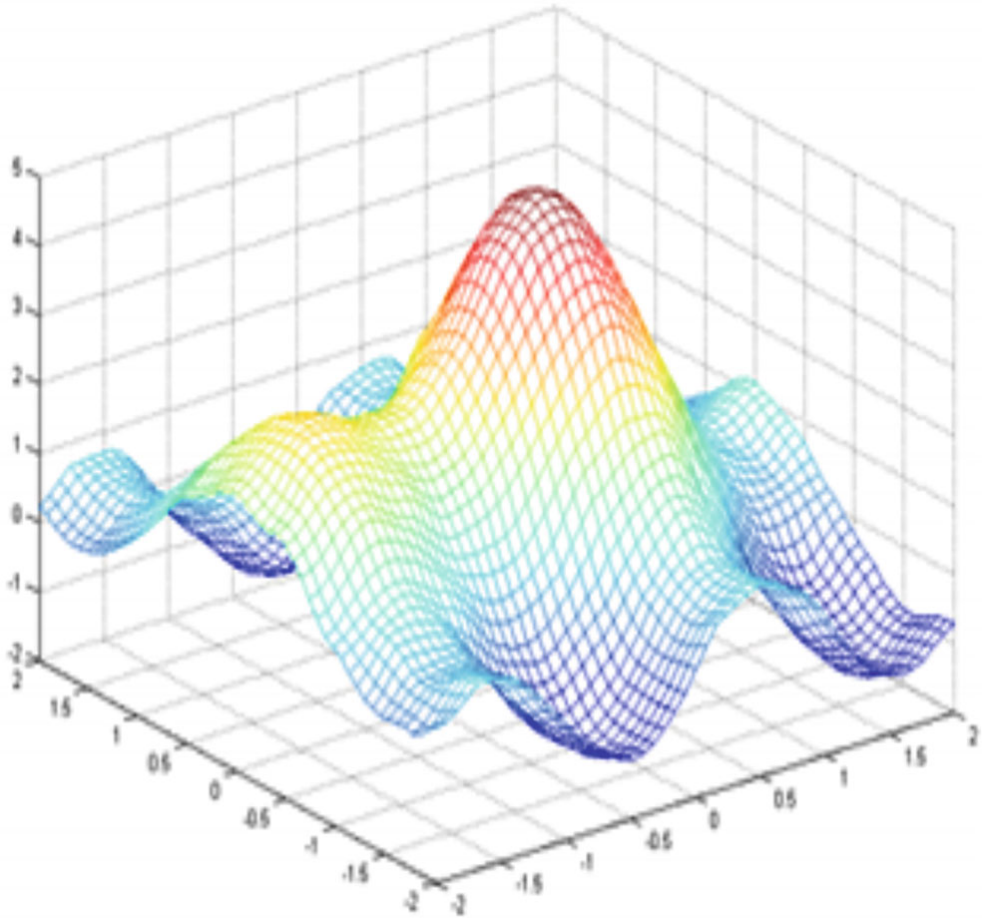
(b)

## 3. SVM Classifier

The main goal is to model the relationship between observations and information to estimate the value of the target for a given prediction base and to understand the relationship between observations and the target. This process is particularly interesting in that it enables automated decision‐making in various fields of application, including medical diagnostics, bank loans, and alarm management. There are many supervised classification methods without a reference one. Thus, to not be dependent on the choice of method in comparing the performance of our attributes, we decided to use the SVM algorithm. *It is an effective* classification technique proposed by Vapnik [65].

The principle of SVM is to represent the inputs of the classifier in a hyperplane (higher dimensional space) according to a nonlinear representation constructed beforehand (learning base). For a two‐class problem, usually, an optimization problem serves as a summary of the idea. Statistics are the basis of the SVM classifier. It is employed in applications when a sizable training dataset is not required for classification. SVM achieves linear discrimination by calculating a hyperplane to divide feature samples into distinct classes.

The hyperplane is computed to maximize the distance between each class’ data samples of marginal features. Therefore, the problem of optimization consists of the following:

i. Identify a hyperplane that will be used to classify samples.
ii. Increase the distance between each class’ marginal feature samples to the maximum. Support vectors, a tiny proportion of marginal samples, offer the solution to this optimization challenge. The decision function (Equation 29) provides the distance of a feature sample from the hyperplane.

(29) f∧x=b+∑i=1naiyiKxi,x,yiє01,,

where *b* is a bias value, *K*(*x*_*i*_, *x*) is a kernel function [66], *x*_*i*_ is the input feature vector, and *i* = 1, 2 ⋯ *n* are the multipliers that are nonzero for the support vectors.

## 4. General Architecture of the Proposed Approach

This method presents a new CAD support system dedicated to mammographic images called MF‐BIMFs, which was based on the combination of three phases: (i) image preprocessing by BMED, (ii) extraction of mathematical formulas from preprocessed images, and (iii) the classification phase. This proposed method will follow the following main steps: The first step, the preprocessing phase, is aimed at improving the image quality before any use. This preprocessing was carried out by the multiresolution decomposition called two‐dimensional empirical mode decomposition (BEMD). Their principle is to decompose the image into several BIMF_*k*_ modes and the residual. It has been used in texture analysis because the extracted BIMFs constitute strong characteristics of the texture. Nunes et al. [27] implemented BEMD for texture extraction, and it was shown that none of the methods, except BEMD, is optimal for all types of images when extracting texture features. This is called a 2D sieving method, such that the envelopes defined by the local maxima and minima, respectively, are locally symmetric around the envelope mean, which defines a BIMF. The decomposition of an image into its BIMFs is called a screening process [27]. The screening process in BEMD decomposition consists of decomposing an input signal into a set of functions defined by the signal itself, called IMFs. An IMF is characterized by specific properties [67].

Each IMF must possess the following properties:

i. The number of zero crossings and the number of extrema are equal or differ by only one unit.
ii. The envelopes defined by the local maxima and minima, respectively, are locally symmetric about the envelope mean.

The screening process stops when the resulting image satisfies the characteristics of an IMF, as described above. In other words, it stops when the mean of the signal envelope is sufficiently close to zero. Once an IMF is found, we define the residual as the result of subtracting this IMF from the input image and then iterate over this residual. The BEMD is complete when the residual, ideally, contains no more extrema. This is valid if we assume that the screening process converges, which has never been rigorously proven. Therefore, a stopping criterion for the screening process must be determined. This can be accomplished by limiting the size of the SD, calculated from the two consecutive screening results, as follows: In practice, we have used a SD between 0.02 and 0.3, and this stopping criterion gives satisfactory results. Generally, the decomposition stops when the number of extrema is less than 2; this means that there are no more oscillations to extract. In some cases, we may have a high number of IMFs without this condition being met. We can stop the decomposition as needed; for example, for image denoising, only the first IMF will be needed. Therefore, it is necessary to set a maximum number of IMFs to extract. In this work, we observe that the decomposition successively extracts the IMFs with the highest local spatial frequencies recursively, leaving these in the residual. The restored image is therefore identical to the input image. Thus, each IMF contains an oscillatory mode inherent to the data at a different spatial frequency range. In order to reduce the number of extracted IMFs, the decomposition was stopped in the first five IMFs for all images in the database. We can then continue the decomposition until we obtain a minimum number of extrema in the residual [28]. The objective of the proposed method is to decompose the image into several modes to find the orientation of the areas of interest of the mammogram of each mode, to eliminate noise, and to improve the image quality. Although BEMD is a useful decomposition tool for texture analysis, it presents the problem of mode mixing: A single BIMF is composed of signals of different scales, or the mode is mixed in another BIMF. In the second step, the extraction of descriptors is aimed at characterizing the lesions through mathematical formulations, so that the result of this step will be a feature vector. In this work, we used multifractal analysis that quantifies fluctuations in the regularity of the texture of images and their local and complex dependency structures. Multifractal analysis is a mathematical technique used to study the scale properties and local variations of functions, measurements, or stochastic processes. It allows the characterization of complex objects, especially those that do not present a single fractal dimension but rather a spectrum of dimensions. In other words, it analyzes how the regularity of an object changes from one point to another. The power of multifractal analysis is discriminating in the separation of pixels belonging to the healthy ones from those concerning the cancerous area for all BIMF modes and the residual. For this, we calculated the parameters based on the Hölder exponent *α* and the multifractal spectrum *f*(*α*) for each pixel of the images for all modes. Finally, the parameters were represented and compared in order to observe if they contribute to improving the discrimination of the groups. For the third step, it is the classification and decision‐making phase using an SVM data classifier. It relies on AI techniques to manage decision‐making.

The BEMD algorithm applies a sieving process to decompose a discrete‐time signal *f* (*x*, *y*) into a set of two‐dimensional IMFs, *f*_*k*_(*x*, *y*), *k* = 1, 2 ⋯ ⋯.*N*, and a residual signal, *R*_*N*_(*x*, *y*), such that (Equation 30):

(30) fkx,y=∑i=1NIMFkfx,y+rNx,y.

We define a source signal (or image), *f* (*x*, *y*), as eligible for BEMD calculation if it has a sufficient number of extremes, at least two: a maximum and a minimum. The process of extracting the intrinsic modes of the signal, namely, the IMF and residual components, using the BEMD algorithm, is described as follows.

– The total number of extrema (minima and maxima) and the number of zero crossings must equal or differ at most by one.
– The mean value of the upper and lower envelopes, at any point, is equal to zero; a stopping criterion, SD, for stopping the sieving process has been mainly proposed as the maximum number of iterations allowed and SDmax, the limit of the SD between two successive results of the sieving process.
  • Initialize the iteration index *j* = 1 and the source image *f* *j* − 1(*x*, *y*) = *r* *j* − 1(*x*, *y*)
  • Estimate the lower EnvMin (*x*, *y*) and upper EnvMax(*x*, *y*)
  • Compute the envelope’s mean

(31) Mj−1x,y=EnvMaxx,y+EnvMin x,y2

• Compute the SD between *f* *j* − 1(*x*, *y*) and *f* *j* (*x*, *y*) with a given image region *f* (*x*, *y*) of sizes *N*_*x*_ and *N*_*y*_

(32) SDj=∑x=1Nx∑y=1Nyfjx,y−fj−1x,y2∑x=1Nx∑y=1Nyfj−x,y2

• Increase the iteration index: *j* = *j* + 1 (apply the sieving steps again)
• If the obtained residue *r*_*k*_(*x*, *y*) is eligible for BEMD, increase the component index BIMF_*k*=*k*+1_ and reapply the sieving process to extract a new IMF. Otherwise, the BEMD algorithm is terminated
• For a decomposed image *I*, it is presented according to the following (Equation 33):

(33) I=∑k=1nBIMFk+Ir,n∈Ν,

where BIMF_*k*_ is the *k*th oscillation, *I*_*r*_ is the decomposition residue, and *n* is the number of BIMFs.

Then, the attribute extraction step was performed to characterize the lesions through mathematical formulations, which are defined as a quantitative measurement or analysis of breast mammographic images. This multifractal analysis allows analyzing fluctuations in the regularity of the texture of the images and distinguished normal and abnormal regions, which quantify their local and complex dependency. In this work, we used the “box‐counting” algorithm, a regular mesh of the image of step *s*, which is the size of the box. The estimation of *α* is done by linear regression of all coordinates. *q*th is the interval of moments of the order of an image. The exponent *α* (*q*, *s*) is given by (Equation 34):

(34) αq,s=limr⟶0∑i=1Nμiq,s×logPiq,slogs.

With *P*_*i*_(*q*, *s*) is the probability of discovering a pixel *i* in a box of fixed size *s*, and *μ*_*i*_(*q*, *s*) is the normalized measure. This step produces feature vectors representing the multifractal analysis measurements of each mammographic region as follows:

(35) α=α0,α1,α2,⋯,αn,

where *n* is the maximum number of BIMF components and *α*_0_ defines the Hölder exponent of the original image.

*α*_*k*_, for *i* = 1, 2, ⋯, *n*, represents the Hölder exponents of the *k*th BIMF_*k*_ component.

Then, the vectors of the pairs ((*α*, *f*(*α*)) and (*Δ* *α*, *Δ* *f*(*α*))) were calculated on each BIMF_*k*_ mode, the residuals, and the reconstructed image and for the original images without preprocessing.

*α* is the Hölder exponent, and *f*(*α*) is the multifractal spectrum.

(36) Δα=αmax−αmin,

(37) Δfα=f αmax−fαmin,

where (*α*_max_) is the maximum value of *α* and *α*_min_ is the minimum value.

The utility of image representation using BEMD for multifractal characterization of distortion is that we apply the proposed methods to distinguish abnormal mammographic regions representing distortions in the center and normal regions representing normal breast parenchyma. These feature vectors formed from the multifractal analysis measurements are then used for the classification of different breast tissue textures and the categorization of healthy and cancerous images with an SVM classifier that was used.

The noninvasive SVM classifier, a well‐known linear classifier with a radial basis kernel function, is used to distinguish patterns of normal and abnormal regions. A classification rate was calculated by this classifier, which will then generate a decision using the selected descriptors. The properties of an SVM‐based pattern classification that is suitable for different pattern recognition problems are as follows: (1) the ability to be trained using a small number of samples and (2) the ability to be applied to solve both single‐class, binary, or two‐class, and multiclass problems. The machine learning approach of SVM classification is derived from modern statistical learning theory. An SVM classifier finds an optimal hyperplane that maximizes the separation (geometric margin) between patterns of different classes [68]. And, at the end, we will make a comparison between the obtained results. The essential phases of the algorithm of the proposed method are described as follows:

*Phase 1*:

i. Determination of the pairs ((*α*, *f*(*α*)) and (*Δ* *α*, *Δ* *f*(*α*))) of all the original healthy and cancerous images without preprocessing.

*Phase 2*: preprocessing. With the BMED method

i. For each *k* = 1–5.

– Extract the *k*th BIMF of all healthy and cancerous individuals.

ii. Determination of the classification rate (%) with the SVM classifier.

*Phase 3*: Determine the pairs ((*α*, *f*(*α*)) and (*Δ* *α*, *Δ* *f*(*α*)(*α*))) of all the healthy and cancerous cases of the reconstructed images: reconstructed images:

(38) Images reconstructed=∑k=15BIMFk+The RESIDU.

*Phase 4*:

i. Determination of the classification rate (%) with the SVM classifier.
ii. Decision‐making.

**Figure 6:**
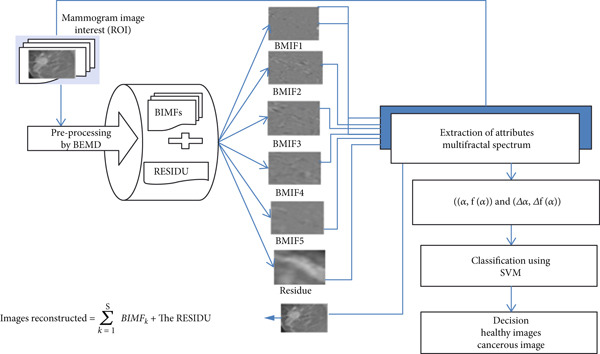
Description of the architecture of the approaches proposed for the diagnosis of breast cancer by the calculation of the multifractal spectrum of the preprocessing images with bidimensional empirical multimodal decomposition (BEMD).

Figure 6 presents the description of the architecture of the approaches proposed.

## 5. Experimental Results and Discussion

### 5.1. Mammogram Datasets

Mammography is a diagnostic radiological procedure used to identify breast diseases. Using x‐rays to create high‐resolution images of the breast, the database contains 260 medical images collected from the Reference Center for Reproductive Health in Kenitra, Morocco. These images are evaluated by experts in the field. This dataset contains 130 mammographic images in their normal forms and 130 images in the cancerous state. From each mammographic image, a region of interest (ROI) of size 400 × 400 pixels with a resolution of 200 pixels is extracted. This ROI represents regions of the true positive (TP), normal breast regions, or true negative (TN). Examples of cancerous and normal ROIs extracted from the database (RCRHKM) are shown in Figure 7, normal ROIs (Figures 7a, 7b, 7c, and 7d), and cancerous ROIs (Figures 7e, 7f, 7g, and 7h). In addition, the important factors impacting the task of mammography are mainly the breast density (in healthy and cancerous mammograms) and subtlety (or visibility) of the lesion and its severity. The ROI should be taken from the whole image to apply the suggested preprocessing and feature extraction techniques in mammographic images; doing otherwise would produce undesirable results. In addition, some tumors overlap normal breast tissue and have low contrast. It is generally accepted that masses with low density, round or oval shape, and well‐defined margin are benign, while masses with high density, spiculated margin, and ill‐defined shape are classified as malignant [69]. The ROI refers to the suspicious mass in the mammographic image that is manually cropped from the preprocessed image in this work. The distribution of breast densities of healthy and cancerous mammographic images, used in this work, is shown in Figure 8. For the cancerous mammograms used, see Figure 8a. The distributions of breast density in the normal case, as well as the distribution of the subtlety and severity of the lesion, are shown in Figure 8b.

Figure 7Some examples of databases from the (a–d) normal ROIs and (e–h) cancerous ROIs.

**Figure:**
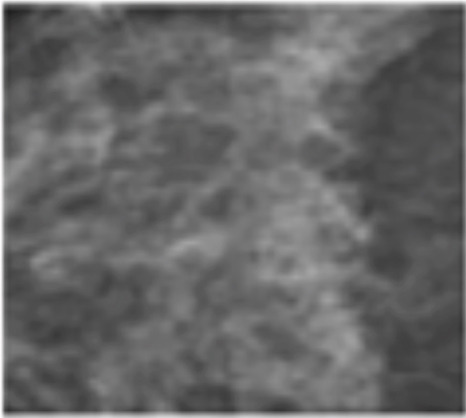
(a)

**Figure:**
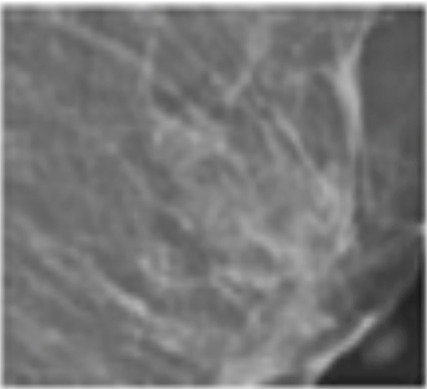
(b)

**Figure:**
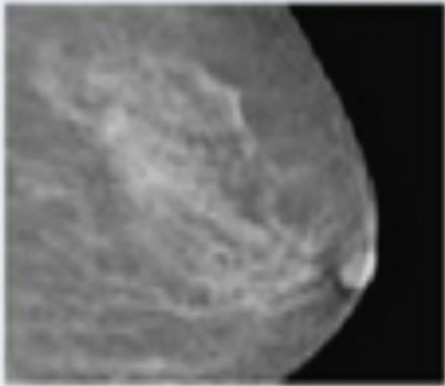
(c)

**Figure:**
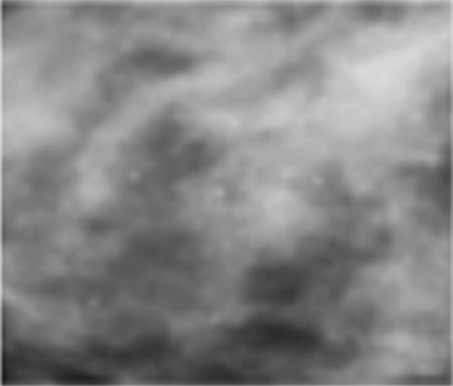
(d)

**Figure:**
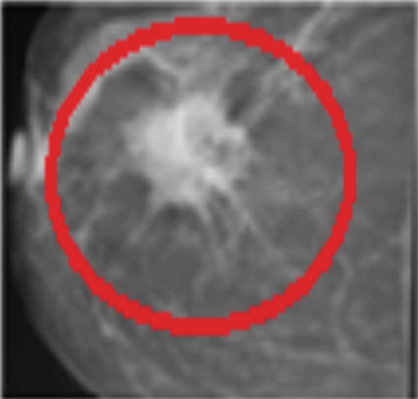
(e)

**Figure:**
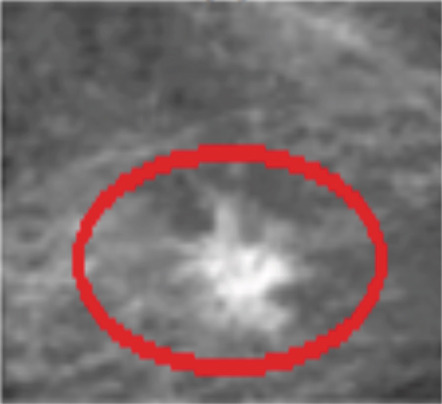
(f)

**Figure:**
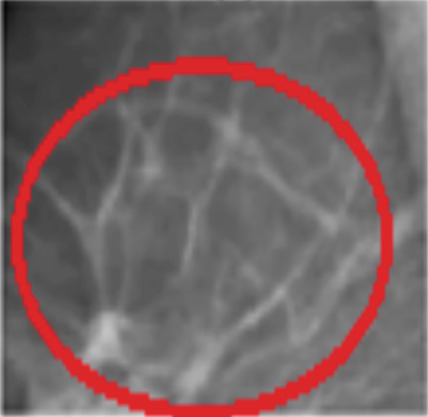
(g)

**Figure:**
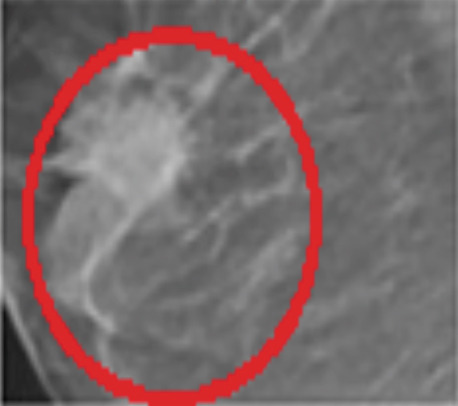
(h)

Figure 8(a) Distributions of breast density, on a scale of 1–4, in normal mammograms. (b) Distributions of cancerous lesions in the mammograms used, in terms of breast density, on a scale of 1–4, of subtlety or visibility of the lesion, on a scale of 1 (*subtle*) to 4 (*obvious*).

**Figure:**
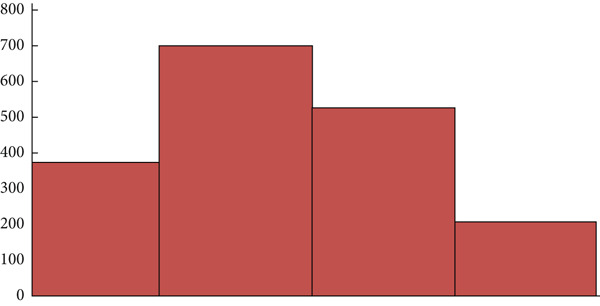
(a)

**Figure:**
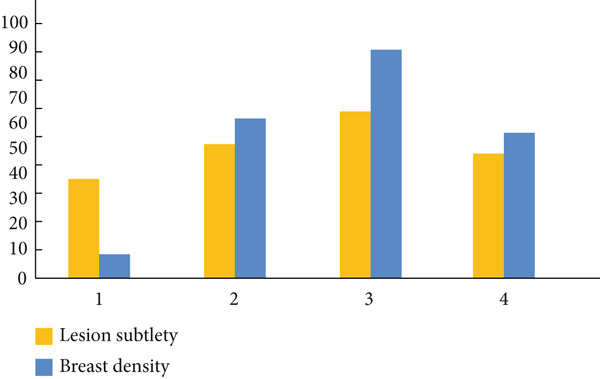
(b)

### 5.2. Preprocessing

Before attribute extraction, we preprocessed mammographic images using BEMD, which consists of decomposing an image into several BIMFs and a residue. The proposed work was tested in a MATLAB 8.0.0 environment on a computer with an Intel(R) Core(TM) i5 processor with a 2.50 GHz CPU and 8.00 GB of RAM.

### 5.3. Extraction of IMFs (BIMFs)

**Figure 9:**
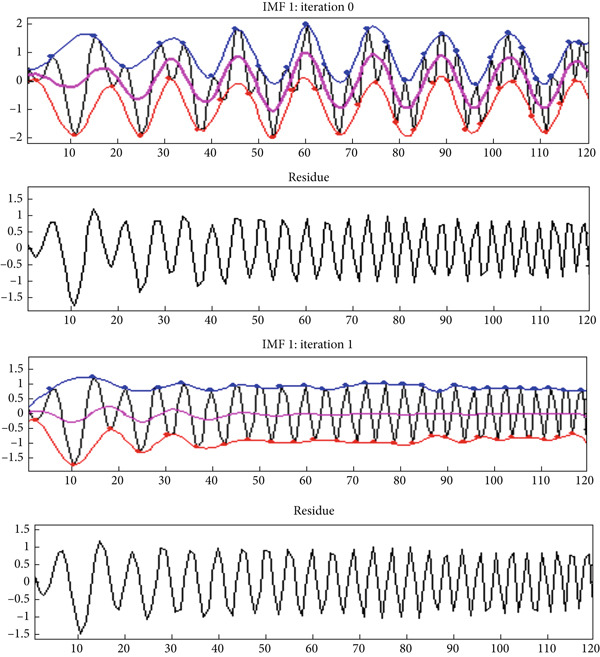
Examples of singular decomposition and the construction of an IMF1 with Iterations 0 and 1.

The result of an EMD decomposition of a two‐dimensional signal *S*(*x*, *y*) for the BEMD decomposition of a mammographic image is shown in Figure 9 which shows the signal decomposition and the construction of an IMF1 with Iterations 0 and 1. When the characteristics of the signal *S* are decomposed, no prior knowledge is present. First, it allows the extraction of high‐frequency components such as BIMF 1 and 2. Starting from the third BIMF, low‐frequency components start to emerge. It can be said that 2D EMD works similarly to a self‐adaptive filter bank, just like 1D EMD. With the help of BMED, the corresponding lower frequencies are obtained. The sieving is repeated several times (*i* times) until a BIMF is obtained. The stopping criterion of the sieving process is based on the properties of BIMF. Moreover, the quality of BIMF extraction depends on the quality of previous BIMFs. There are situations in which we can have a large number of BIMFs without this condition being met. The choice of this maximum number is linked to the specificities of the applications. Several experiments were performed by varying the maximum iteration, the stopping criterion of the sieving process, and the size of the filter used for envelope estimation. We empirically chose to use the IMF decomposition parameters with a maximum number of iterations to extend an image along the boundaries needed to filter images with a finite representation. For an efficient BEMD analysis, EMD research studies have suggested that a sieving process with a large number of iterations or very small SDmax values should be applied [8]. This sieving process was mainly proposed to refine the extraction of IMF components such that each BIMF meets the following two requirements:

– The total number of extremes (minima and maxima) and the number of zero crossings must be equal or differ by at most one.
– The average value of the upper and lower envelopes, at any time, is equal to zero.

**Figure 10:**
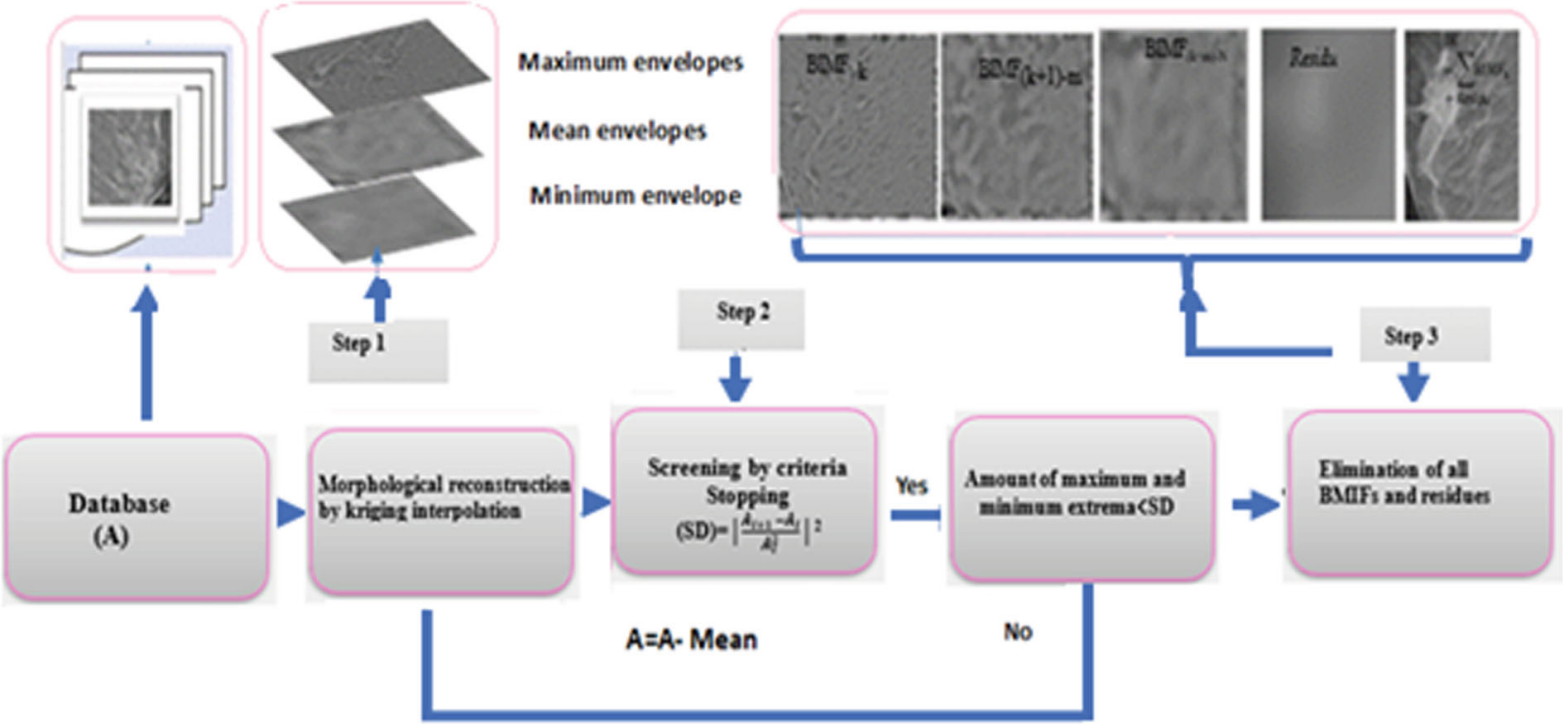
Schematic diagram of the bidimensional empirical mode decomposition (BEMD) algorithm for breast mammography image.

**Figure 11:**
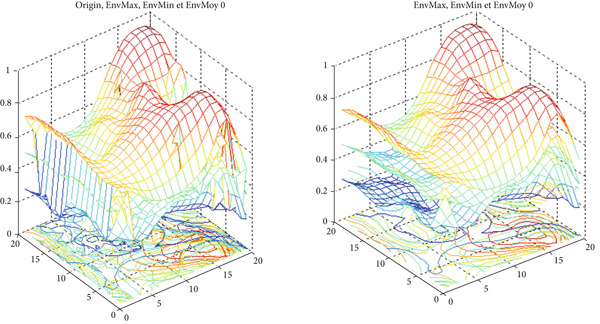
Example of the construction of maximum (EnMax) and minimum (EnMin) envelopes and the mean (EnMoy) of a mammographic image.

**Figure 12:**
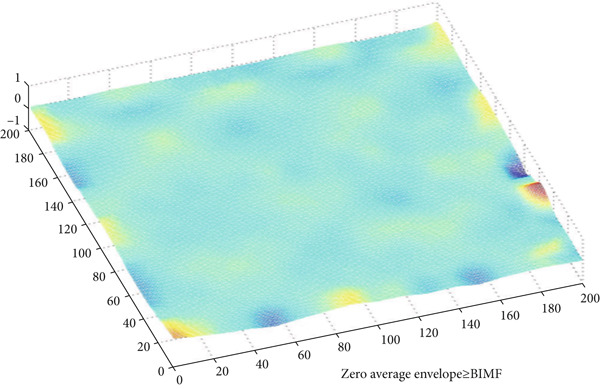
Example of the construction of the BIMF of a mammographic image.

**Figure 13:**
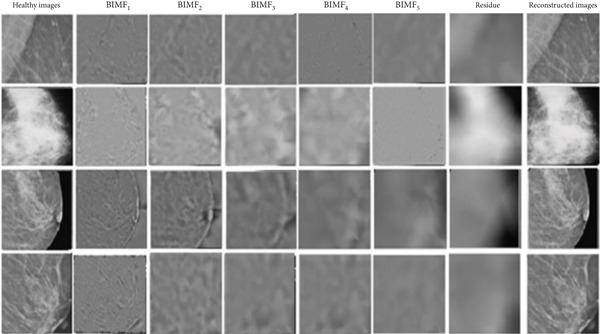
Examples of healthy images preprocessed by the empirical two‐dimensional multimodal BEMD decomposition into five levels of BIMF_*k*=1⋯5_ and the residuals and reconstructed images.

**Figure 14:**
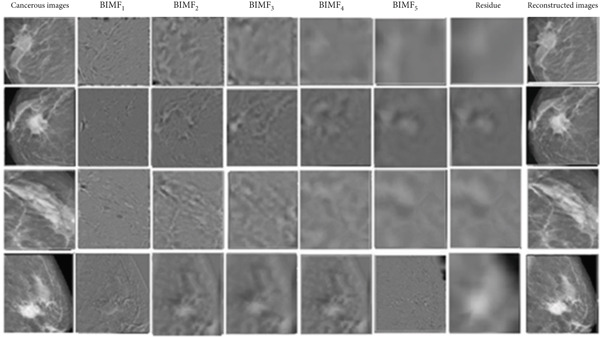
Examples of cancerous images preprocessed by the two‐dimensional multimodal empirical decomposition (BEMD) at each level of the BIMF_*k*=1⋯5_ and the residuals and the reconstructed images.

Figure 10 illustrates the steps of BEMD decomposition for a breast mammographic image. Figures 11 and 12 show an example of constructing the maximum and minimum envelopes and the mean and constructing the BIMF of a mammographic image, respectively. Most images produce a BEMD decomposition with five BIMF_*k*_. In this study, five will be used for image analysis of the datasets (RCRHKM). The examples of the BEMD algorithm applied to abnormal and normal mammographic regions are shown in Figures 13 and 14, respectively. The healthy and pathological images are decomposed at five levels of BIMF = 1, 2, 3, 4, and 5, as well as the residuals and reconstructed images. To ensure that the pattern representation and thus the number of features obtained are the same for all images, the number of IMF components common to all images in our datasets is used for feature extraction and pattern classification.

### 5.4. Feature Extraction

**Figure 15:**
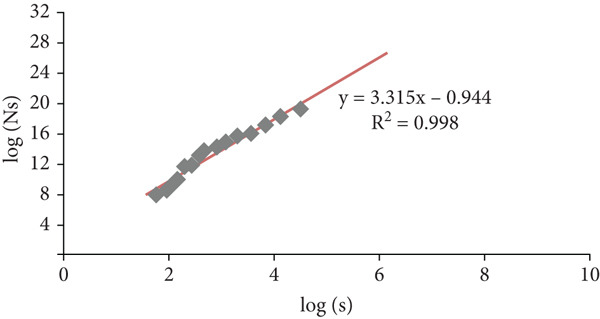
Linear regression of log(Ns) as a function of log(*s*) for two sets of box sizes *s*: *s* = {2, 4, ⋯, 64}.

In this work, we used the “box‐counting” algorithm to calculate the multifractal attributes. The initialization parameters for this algorithm are the interval of box sizes *s*. A way must be found to efficiently choose the two values, namely, *s*_min_ and *s*_max_. For this, we used the essential step of simple linear regression (SLR). SLR is particularly sensitive to “outliers,” especially if it is not performed on a large number of observations. When outliers are likely to distort the equation of the regression line, it is preferable to exclude or impute them. Moreover, it is easy to quantify the accuracy of the regression by calculating the coefficient of determination (*R* ^2^). Therefore, we tested different values of *s*_min_ and *s*_max_ and retained those for which the coefficient of determination was the highest. Figure 15 shows the best *R* ^2^ coefficient of the linear regression of 4, log(Ns) as a function of log(*s*) for two sets of sizes *s* of the boxes *s* = {2, ⋯}. On the one hand, we noticed that the *R* ^2^ is very high (*R* ^2^ = 0.992), which confirms the applicability of the calculation of multifractal attributes by this method on images. In conclusion, for the “differential box‐counting” method, the size interval retained is {2, 8, 10, …, 64}.

**Figure 16:**
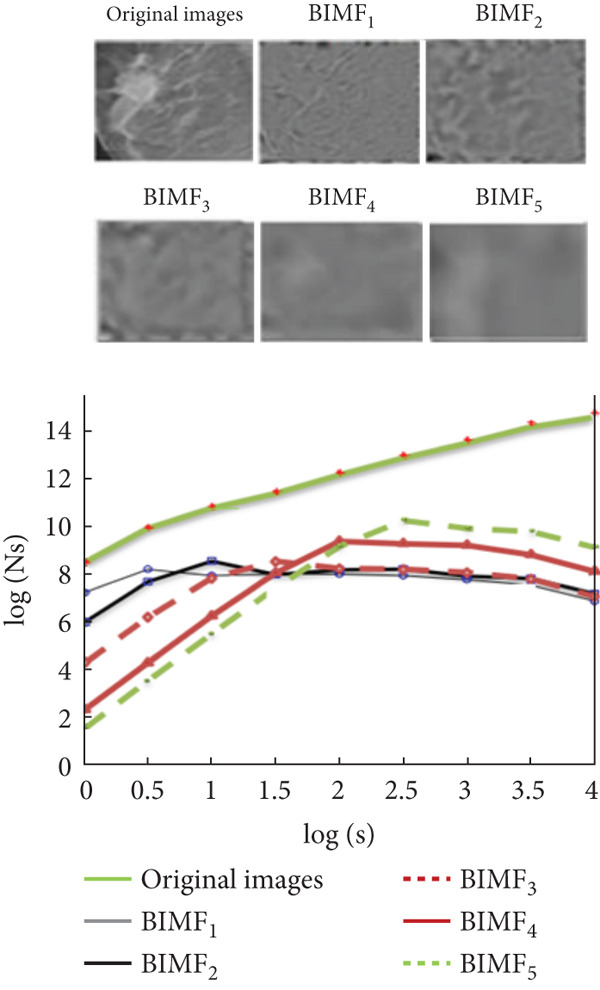
Example of the Hölder exponent function measurements of an original cancer region and its BIMF_*k*=1⋯5_ obtained from BEMD.

An example of the Hölder exponent function measurements of an original cancer region and its BIMF_*k*_ obtained from BEMD analysis from the dataset is shown in Figure 16. The Hölder exponent function of each image is then calculated from the linear fit of the corresponding log(Ns) plot. The intricacy and regularity of the breast in this instance are represented by the multifractal spectrum *f*(*α*). When evaluating breast tissue quantitatively during mammography, multifractal characteristics seem to be useful biomarkers. Equations (11) and (12) were used to determine the spectrum from the image extracts. The multifractal spectrum is calculated using two additional parameters following the pretreatment process. Following multiple experiments utilizing a feature vector made up of multifractal analytics descriptors, *r* varied between 2 and 64 with a step of 2 and was shown to be sufficient and beneficial for improving population categorization.

When examining the texture of an image, the interval of moments of order *q*th can be compared to a potent microscope. After several experiments, we found that the interval confirms the linear behavior of the functions given by Equations (11) and (12) of −4 < *q* < 4 with a step of 0.1. The multifractal behavior of breast mammography images is properly highlighted by this period. It is possible to derive additional features from the multifractal spectrum’s form. Specifically, the multifractality degree of the samples is determined by the breadth of the spectrum (*Δ* *α* = *α*_max_ − *α*_min_). The width of the multifractal spectrum *Δ* *f*(*α*) is calculated by the following equation *Δ* *f*(*α*) = *f* (*α*_min_) − *f*(*α*_max._) where *α*_max_ is the maximum Hölder exponent and *α*_min_ is the minimum value.

**Figure 17:**
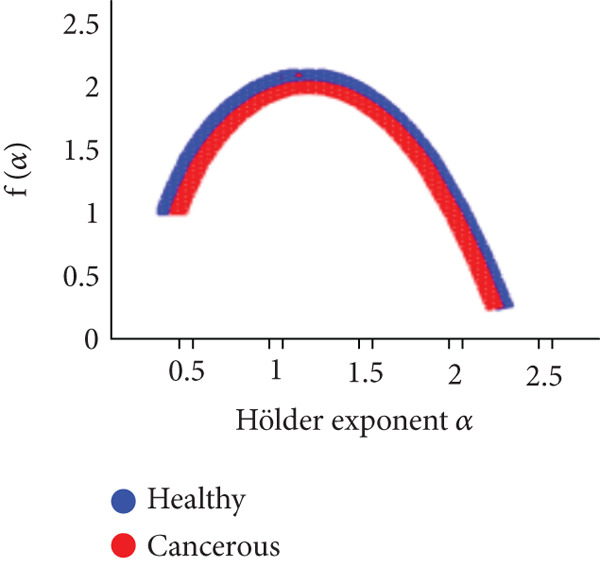
Multifractal spectrum of pathological (red) and healthy (blue) cases.

For each group of photos (healthy and malignant), direct computation of each of these parameters was done using the multifractal spectrum. We investigated the attribute’s discriminative capacity in the separation of voxels belonging to the healthy ones from those not cancerous in the peripheral zone in order to assess the attribute’s efficacy based on the multifractal spectrum. For this calculation, we looked at mammograms of the original images and each BIMF, residual images, and rebuilt images of its figure (healthy and cancerous). We also calculated the pair (*α*, *f*(*α*)), *Δ* *α*, and *Δ* *f*(*α*). To see if these parameters aid in better group discrimination, they were represented and compared in two groups. The results are displayed in Table 1. Figure 17 presents the variation of the Hölder exponent function order *q*th and of the multifractal spectrum (*α*).

**Table 1:** Variation of parameter values: the Hölder exponent *α*, the multifractal spectrum (*Δ* *α* = *α*_max_ − *α*_min_ ), and *Δ* *f*(*α*) = *f* (*α*_max_) − *f*(*α*_min_) for each mode BIMF_*k*=1⋯5_, residuals, reconstructed images (Rec_image), and original images (Origi_image) of the dataset: pathological (130 cases) and healthy (130 cases).

**Number of ROI**	**Method**	**BIMFs**	**α**		**f** (**α**)		**Δ** **α**		**Δ** **f**(**α**)	
			**Pathological**	**Healthy**	**Pathological**	**Healthy**	**Pathological**	**Healthy**	**Pathological**	**Healthy**
– 130 of normal ROI – 130 of cancerous ROI	**MF-BIMFs**	**BIMF**_**1**_	1.391*%* ± 6.33*%*	2.201*%* ± 2.05*%*	1.481*%* ± 1.06*%*	0.91 ± 0.003	0.904 ± 0.003	0.902 ± 0.002	0.018 ± 0.09	1.391*%* ± 6.33*%*
		**BIMF**_**2**_	1.052*%* ± 9.3*%*	1.345*%* ± 3.85*%*	1.676*%* ± 4.01*%*	0.730 ± 0.152	2.022 ± 0.104	0.028 ± 0.089	0.979 ± 0.189	1.052*%* ± 9.3*%*
		**BIMF**_**3**_	0.365*%* ± 7.05*%*	1431*%* ± 3.3*%*	1.768*%* ± 4.3*%*	0.520 ± 0.022	0.491 ± 0.018	0.089 ± 0.095	2.028 ± 0.089	0.365*%* ± 7.05*%*
		**BIMF**_**4**_	0.771*%* ± 1.06*%*	1.464*%* ± 5.65*%*	1.834*%* ± 2.5*%*	0.504 ± 0.032	0.471 ± 0.039	0.494 ± 0.014	0.492 ± 0.017	0.771*%* ± 1.06*%*
		**BIMF**_**5**_	1.711*%* ± 1.06*%*	0.764*%* ± 5.65*%*	0.96*%* ± 2.25*%*	4.606e − 06	7.566e − 08	0.468 ± 0.030	8.622e − 11	1.711*%* ± 1.06*%*
		**Residue**	0.235*%* ± 5.04*%*	0.47*%* ± 55*%*	0.956*%* ± 5.5*%*	0.504 ± 0.032	0.471 ± 0.039	0.504 ± 0.032	0.471 ± 0.039	0.2356*%* ± 5.04*%*
		**Rec_image**	0.793*%* ± 93*%*	0.41*%* ± 8.3*%*	0.773*%* ± 8.3*%*	0.471 ± 0.039	5.606 ± 03	0.35*%* ± 3.85*%*	0.54*%* ± 1.65*%*	0.793*%* ± 93*%*
		**Origi_image**	0.204*%* ± 19.4*%*	0.168*%* ± 2.5*%*	0.574*%* ± 6.35*%*	0.464*%* ± 5.35*%*	0.364*%* ± 5.65*%*	0.664*%* ± 4.65*%*	0.37*%* ± 25*%*	0.204*%* ± 19.4*%*

## 6. Classification Results

To quantitatively evaluate the above observations, the nonlinear SVM classifier is trained on our training set. The nonlinear SVM kernel and RBF options were used. A grid search with a *C* value ranging from 0.5 to 1,156,666 and a kernel control parameter value *γ* ranging from 0.0722 to 16 was used for the SVM penalty (or regularization) constant and Gaussian kernel control parameter, respectively.

### 6.1. Statistical Analysis

To evaluate the effectiveness of the multifractal spectrum, we defined predicted demand and studied its discriminative power in the separation of pixels belonging to healthy ones from those considered in the peripheral zone for all BIMF modes and the residue. For this, we calculated the parameters based on the multifractal spectrum as described previously for each pixel of the images for all modes. Finally, the parameters were represented and compared to observe if they helped to improve the discrimination of the groups. For this reason, we took 22 values for each of the two sets that were calculated, corresponding to the 44 patients in our database. These values were represented as scatterplots, where the points (“blue”) represent the attribute values for the voxels belonging to the healthy individuals, while the cross points (“red”) correspond to the cancerous ones. Figure 18 shows the cloud of points obtained for the BIMF modes, the residual, reconstructed image, and the original image from the results obtained. We note that for BIMF_1_ and BIMF_2_, the multifractal attribute is better able to discriminate between the two sets. Indeed, if we project the points on one of the two axes, we see that the classification error is better, there is greater interest, and there is better efficiency of our procedure for estimating the Hölder exponents for the first two modes. Even if the characteristic measured for the BIMF_1_ mode seems more effective than that obtained by BIMF_2_, the first important point is that there is no correlation between the two attributes, which justifies the choice to combine them. Furthermore, given the point cloud in Figure 18a,b, we have seen that it seems quite effective in discriminating voxels belonging to the tumor from those not belonging to it; the mode seems more efficient than that obtained by BIMF_2_. The first important point is that there is no correlation between the two attributes, which justifies the choice to combine them. Furthermore, given the point cloud in Figure 18a, we have seen that it seems quite effective in discriminating voxels belonging to the tumor from those not belonging to it. For the other modes, Figures 18c, 18d, 18e, and 18f are less discriminating. The multifractal attribute of healthy images exists in the range of pathological images and vice versa, and the variation intervals of these parameters overlap. For the reconstructed images (Figure 18g) and the original images (Figure 18h), the correlation can be explained by the fact that they both characterize the same information. We note that we do not make it possible to establish a differentiation between the two populations.

Figure 18Representation of (a–e) multifractal spectrum in point cloud for BMIF_*k*_ = (1, 2, 3, 4, 5), respectively, (f) for the residual, (g) for the reconstructed, and (h) for the original image.

**Figure:**
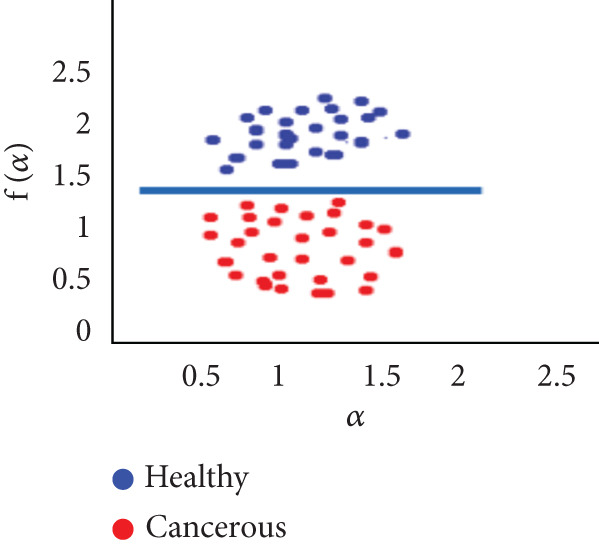
(a)

**Figure:**
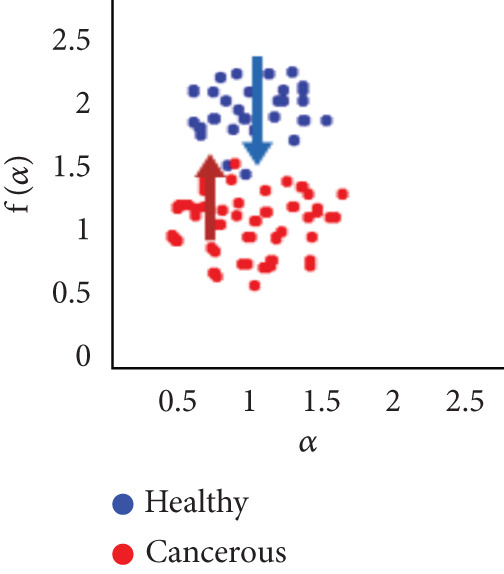
(b)

**Figure:**
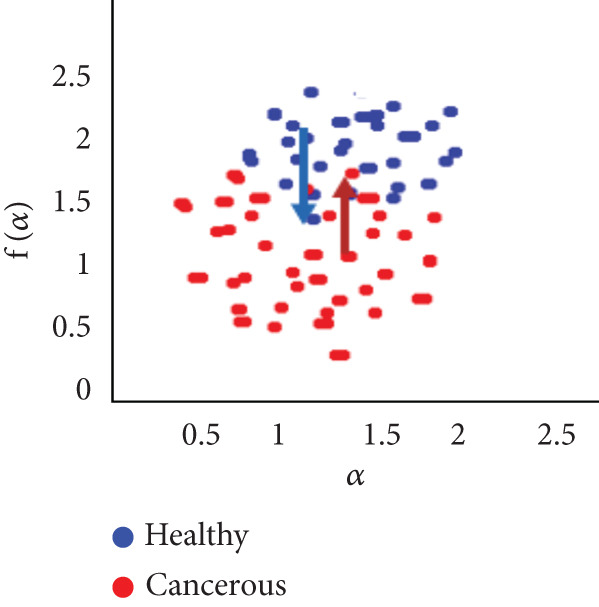
(c)

**Figure:**
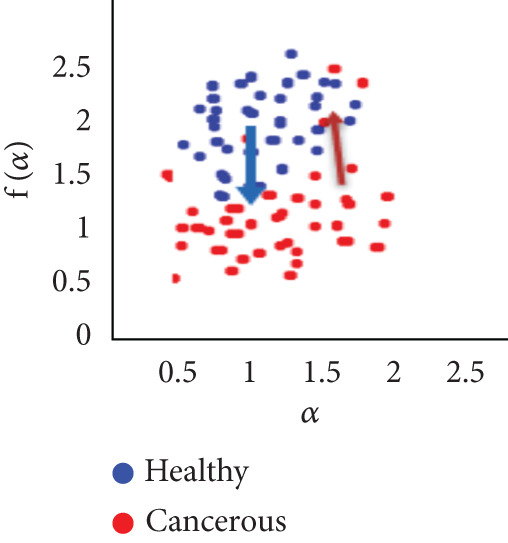
(d)

**Figure:**
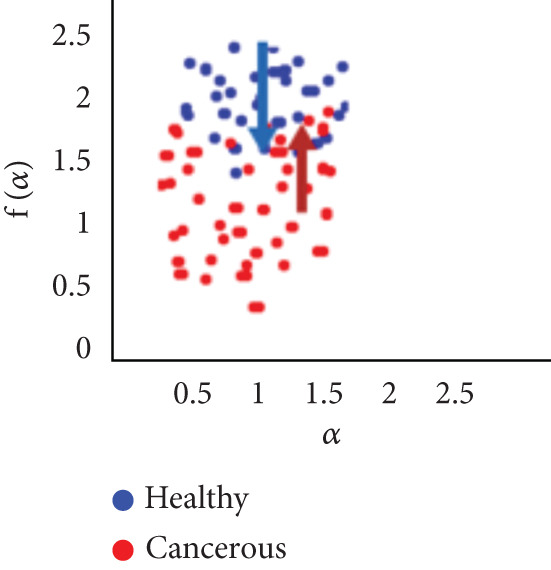
(e)

**Figure:**
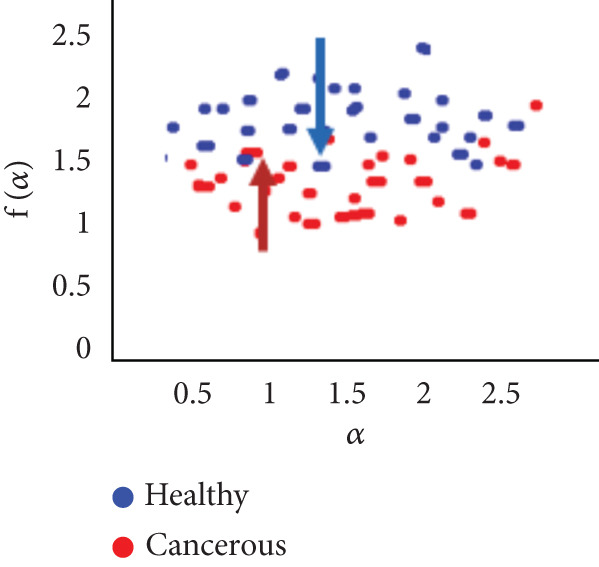
(f)

**Figure:**
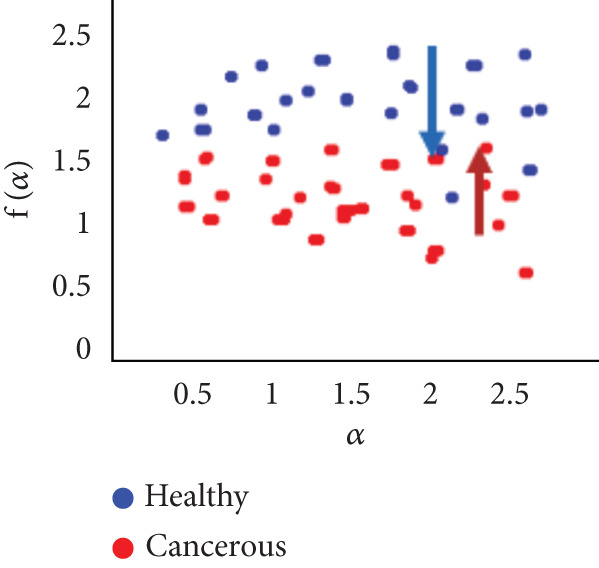
(g)

**Figure:**
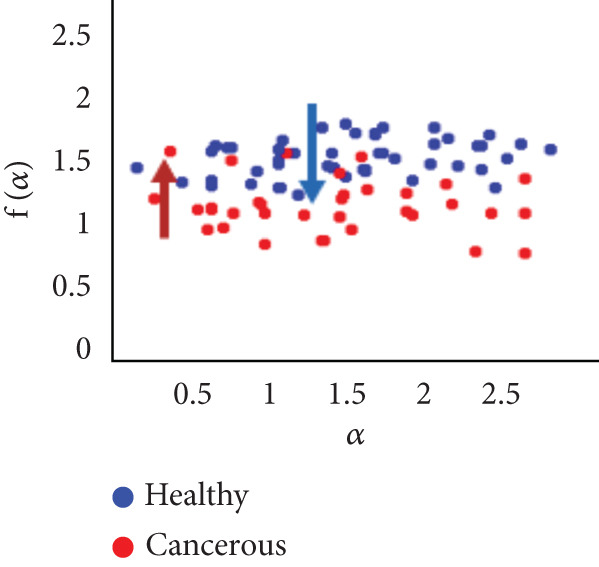
(h)

It seems impossible to determine the two sets of points (“tumor”/“nontumor”).

### 6.2. Analysis of Accuracy, Sensitivity, Specificity, Precision, F1‐Score, and AUC

To evaluate and compare the effectiveness of various methods, we will use seven metrics to evaluate the performance of different models: accuracy, sensitivity, specificity, precision, F1‐score, and AUC. The formulas for each evaluation metric are shown in Table 2.

– TP means that the predicted result of the classifier is positive and the actual sample is positive, that is, the number of positive samples correctly identified.
– False negative (FN) means that the predicted result by the classifier is positive samples, but they are negative samples, that is, the number of negative samples that are wrongly predicted as positive.
– TN means that the predicted result by the classifier is negative samples, but they are positive samples.
– FP refers to the number of negative samples identified when the classifier predicts a positive sample.
– Precision can be defined as the proportion of samples that are accurately predicted of the total number of samples. Whether the sample is benign or malignant, it shows the performance and accuracy of the categorization.
– Sensitivity represents the percentage of cases in which a malignant tumor is correctly diagnosed as such. The ability to identify malignant tumors is stronger when the sensitivity is high.
– Specificity is the percentage of cases of samples with benign tumors that are accurately classified as benign. The ability to identify benign tumors is stronger when the specificity score is high.
– The accuracy of predicting correct positive samples. Higher precision means fewer false detections, and lower precision means more false detections.
– The F1‐score is based on precision and recall (sensitivity). Its main objective is to maximize both accuracy and sensitivity while minimizing the difference between these two values.
– AUC is a reliable way to quantify overall quality. The ideal classifier has an AUC of 1 and can accurately identify all categories under any threshold. When AUC = 0.5, the classification is equivalent to random prediction, in which case the classifier is unavailable. It is superior to random prediction when 0.5 < AUC < 1 and is also the state of most classifiers in real‐world applications. It is worse than random prediction when AUC < 0.5.

**Table 2:** Evaluation metrics in our different experiments: sensitivity, specificity, precision, accuracy, and F1‐score.

Sensitivity	$\frac{\text{TP}}{\text{TP}+\text{FN}}$
Specificity	$\frac{\text{TN}}{\text{TN}+\text{FP}}$
Precision	$\frac{\text{TP}}{\text{TP}+\text{FP}}$
Accuracy	$\frac{\text{TP}+\text{TN}}{\text{TP}+\text{FN}+\text{FP}+\text{TN}}$
F1‐score	$\frac{2\times \text{precision}\times \text{sensitivity}\;}{\text{precision}+\text{sensitivity}}$

### 6.3. Analysis of FN and FP Result

**Figure 19:**
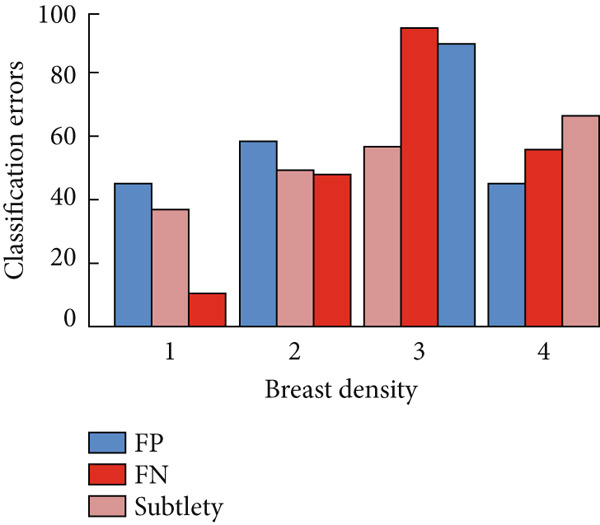
Evaluation of classification errors for the distributions of false positive (FP) and false negative (FN) according to breast density in a scale of 1–4, the evaluation of FN, and subtlety.

Regarding the density of mammograms, whether normal or cancerous cases, the main elements are the subtlety (or visibility) and severity of the abnormality. Factors influencing the choice of mammography equipment during screening mammograms are also taken into account. Following the analysis of these factors, we evaluated the results from the classification carried out. In this study, we use Figure 19 to demonstrate the evaluation of classification errors related to FPs and FNs according to breast density, as well as the appreciation of FNs and their subtlety. The analysis highlights the effectiveness of multifractal measurements. Regarding cancerous and normal areas, they were affected by density. In other words, mammograms with higher density scores (3 and 4) were frequently misclassified, especially those with a density score of 3. Regarding cancer areas, classification and associated FN results, as well as breast density, lesion enhancement, and severity, are key factors to consider that could impact detection efficiency. Examination of the data reveals that the more subtle the lesion, the greater the risk of misclassification, particularly for this study. However, areas with a subtle score of 2 were more often misclassified compared to those with scores of 1 and 3. Analysis of the FN results about lesion severity revealed that areas with a higher severity (higher assessment score), particularly the abnormal area with an assessment score of 4, were often misidentified compared to other scores.

The Hölder exponent *α* between regions without pretreatment and regions with BMED pretreatment is significant: The BIMF_*k*_ regions in the cancerous case have lower *α* values than the BIMF_*k*_ regions in the normal case. Our characterization approach, after applying the BEMD algorithm to produce a multiresolution representation of the breast region, extracted multifractal measures to characterize the breast texture.

Our results were more effective with the parametric statistical modeling of BEMD, which produced an area under the characteristic curve of 0.982 at the BIMF_1_ level. In this paper, the BEMD algorithm was used to provide an adaptive and data‐driven multiresolution representation of breast texture. The variation of multifractal analysis on different scales, based on BEMD, was used to characterize different types of breast texture. Therefore, the variation of multifractal analysis on different ROIs of the images was demonstrated as an effective tool for texture pattern discrimination. The suggestion made above by observing the point clouds appears, with the estimation of the multifractal spectrum in BIMF_1_ mode, to be more discriminating, which is quantitatively justified in this experiment.

**Figure 20:**
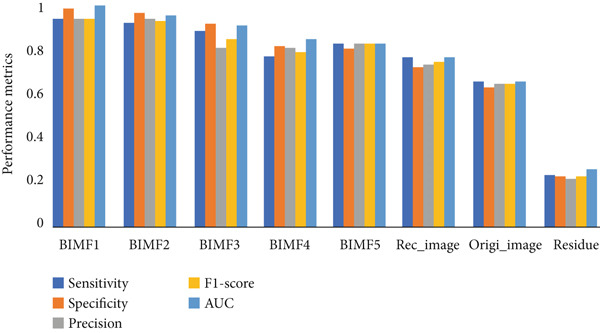
Comparison of the evaluation metrics: sensitivity, specificity, precision, F1‐score, and AUC of various BIM_*k*_ (MF‐BMED), residuals, reconstructed image (Rec_image), and original image (Origi_image) of the multifractal spectrum features with SVM.

**Figure 21:**
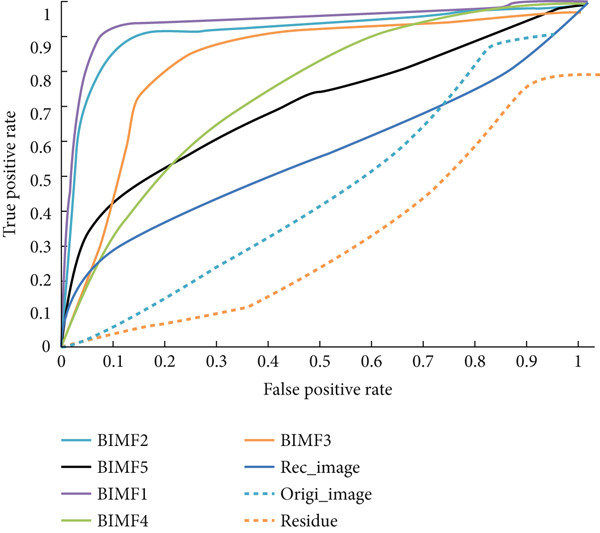
Comparative analysis of the ROC curves between BIMFs (MF‐BMED), reconstructed image (Recn_image), original image (Origi_image), and residual of the multifractal spectrum features with SVM.

**Figure 22:**
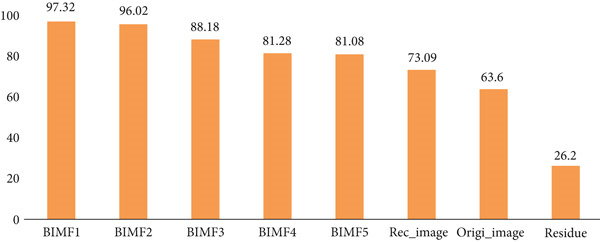
Comparison of the classification performance values between BIMF_*k*_ (MF‐BMED), reconstructed image (Rec_image), original image (Origi_image), and residue of the multifractal spectrum features with SVM.

Table 3 and Figure 20 present and compare the values, classification rate, sensitivity, specificity, accuracy, F1‐score, and AUC of various BIMFs (MF‐BMED), residuals, reconstructed images (Recn‐images), and original images (Ori images), and Figures 21 and 22 represent the ROC curves and performance comparison of BIMF_*k*_ modes.

**Table 3:** Classification of performance metrics, such as sensitivity, specificity, precision, accuracy, F1‐score, and AUC, is assessed for BIMF_*k*=1⋯5_, residuals, reconstructed images (Rec_image), and original images (Origi_image). The highest value is shown in bold.

**Method**	**BIMFs**	**Accuracy classification (%)**	**Sensitivity**	**Specificity**	**Precision**	**F1-score**	**AUC**
MF‐BIMFs	BIMF_1_	**97.32**	0.917	0.963	0.926	0.921	0.982
	BIMF_2_	96.02	0.905	0.944	0.915	0.909	0.956
	BIMF_3_	88.18	0.87	0.894	0.791	0.828	0.89
	BIMF_4_	81.28	0.755	0.795	0.788	0.777	0.825
	BIMF_5_	81.08	0.808	0.788	0.818	0.812	0.815
	Residue	26.2	0.223	0.208	0.203	0.214	0.25
	Rec_image	73.09	0.745	0.705	0.713	0.728	0.741
	Origi_image	63.6	0.641	0.61	0.622	0.631	0.64

The results obtained in Table 3 and Figure 20 show that the evaluation measures of the BIMF_1_ mode outperform all other modes, the images without preprocessing, and the reconstructed images. By observing the values in Table 3 and Figures 21 and 22 which represent the ROC curves and the AUROC values corresponding to the classification rates, we observe that the remarks made above are justified here, such that the classification performance obtained with the BIMF_1_ modes is the most discriminating (AUROC = 0.982), followed by BIMF_2_ (AUROC = 0.956) and the other adjacent modes, and also followed by the reconstructed images (AUROC = 0.741), the original images (AUROC = 0.64) and finally for residual (AUROC = 0.25).

With high visual similarity, new texture properties showing changes in the breast were extracted using a multifractal spectrum. The BIMF_1_ mode enabled these two groups to be distinguished by new texture properties, highlighting changes in the breast that were extracted using a multifractal spectrum. The BIMF_1_ mode allows these two groups to be distinct from each other, so it is possible to define a discrimination threshold between these two groups. It is observed that for the other modes, when the variation intervals of the images (pathological/healthy) are combined, it is not possible to distinguish the two populations.

Image preprocessing and modeling using BEMD are decomposition techniques that decompose an image into a collection of two‐dimensional intrinsic mode functions (BIMFs).

These extracted BIMFs constitute the strongest features of breast mammographic image elements that have significant spatiotemporal variability. Multifractal analysis of these BIMFs is a suitable framework, especially when considering the nonuniformity features of textures. It provides an effective synthetic modeling for discrimination into two healthy and cancerous populations of the process to be analyzed. The obtained results show that there is an improvement in breast cancer diagnosis using our proposed novel approach to classify cancerous and healthy mammographic images.

The classification of BIMF_1_ features has a high accuracy (97.32%), compared to other modes. The extraction of the descriptor vector depends on the spatial dimension of the correlation of the extrema of each intrinsic two‐dimensional modal function (BIMFs), which is extracted by the BEMD. Thanks to this extraction using the depth level, we were able to obtain encouraging results that helped us to diagnose the disease at an early stage. Then, the results obtained are robust and very encouraging.

The performance of the system is relatively consistent when using preprocessed mammograms and also using a dataset from the RCRHKM, which includes normal and abnormal mammograms, adds credibility to the study results, and ensures their relevance for real‐world clinical applications. The results of our proposed work have been compared with the results obtained with the public databases of breast mammography images used in the literature, preprocessed by BMED; for example, the work titled “Feature Extraction Based on Empirical Mode Decomposition for Automatic Mass Classification of Mammography Images,” which was based on gray level co‐occurrence matrix (GLCM) Feature Extraction [29], was performed after preprocessing by BMED and modified MBMED. This work used three real databases, which were collected from the Mammographic Image Analysis Society (MIAS) database and the Digital Database for Screening Mammography (DDSM), which are the publicly available databases, and the images were also collected from the Department of Radiology, Mahatma Gandhi (MGM) Hospital, affiliated to KAPV Government Medical College, Tiruchirappalli, India. The results obtained by using the SVM classifier with BMED are an AUC of 0.9, 0.90, and 0.856 for the MIAS, DDSM, and MGM bases, respectively, and with MBMED are an AUC of 0.966, 0.933, and 0.92 for the MIAS, DDSM, and MGM bases, respectively. By using the LDA classifier with BMED, the AUCs are 0.89, 0.866, and 0.83 for the MIAS, DDSM, and MGM bases, respectively, and with MBMED, the AUCs are 0.933, 0.9, and 0.9 for the MIAS, DDSM, and MGM bases, respectively.

In the work entitled “Computer‐Aided Detection of Architectural Distortion in Digital Mammograms Using BEMD Fractal Dimension Measurements” [30], BMED processing was performed, and fractal dimension extraction was done with an SVM classifier. The performances were obtained at 0.964 and 0.95 for MIAS and DDSM databases, respectively. Our work was used on a real database of RCRHKM, and an AUC of 0.982 was obtained, so we have an improvement in the performance of classification and discrimination between healthy and cancerous images.

This work presents an innovative approach in the field of mammographic image classification based on multifractal analysis of BIMF, establishing a high performance for research in the field of medical imaging, based on the preprocessing of mammographic breast images decomposed by BMED; the future of AI in breast cancer screening is bright, as the increasing use of AI to analyze mammograms and tomosynthesis shows promise. Research is underway to expand its use, particularly for high‐risk women. In the long term, AI could also help determine which patients need chemotherapy; the rapid development and implementation of AI has the potential to revolutionize healthcare. However, to do this, staff must be skilled and confident in its application, so AI readiness is an important precursor to its adoption. Research into how best to deliver this AI‐based medical training is in its early stages.

### 6.4. Comparison With Other Existing Methods

In this section, we compare the classification performance of our proposed method with other existing methods. Table 4 presents a comparison based on performance, publication year, classification algorithms, and dataset. The results in Table 4 indicate that various techniques are used to extract texture features at different orientations: Gabor wavelets, contourlet transform, GLCM, fractal dimension, multifractal measures, and combination with the BMED model and MRF. For example, with the SVM classifier, the accuracy of a work established on BEMD preprocessing is 90%; with MBEMD preprocessing, it is about 92.59%; the distribution of the multifractal spectrum is about 94.8%; and with BMED preprocessing followed by fractal dimension extraction, which reaches a rate of 95%, there is a remarkable improvement for our method (MF‐BMED), which reaches a rate of 97.32%, significantly higher than other breast cancer diagnosis methods and several state‐of‐the‐art works. Increases of 7.32%, 4.73%, 2.52%, and 2.32%, respectively, are remarkable. The difference between the proposed MF‐BIMF approach and existing methods lies in the combination of the multifractal spectrum with a preprocessing technique called BMED to improve the image quality, making it an excellent image preprocessing tool. Two‐dimensional empirical decomposition is an effective method for extracting detailed information from images, thus paving the way for multiscale processing and analysis.

**Table 4:** A comparison of the classification performance of our proposed methodology with other existing methods in the literature is presented in terms of performance, publication year, classifiers, and dataset.

**Existing methods in the literature**	**Performance (%)**	**Years**	**Classifier**	**Feature**	**Database**
Fractal dimension by DBC [40]	50.4	2009	SVM	Fractal dimension by DBC	CHRU lille France
Multifractal measurement [42]	55.1	2009	SVM	Multifractal measurement	CHRU lille France
Multifractal measurement by ref‐mBm [40]	72	2009	SVM	Hölder with ref‐mBm	CHRU lille France
LBP combined with a uniform model for feature extraction and SVM [45]	74	2016	SVM	LBP	DDSM
Gradient analysis for classification of mammographic masses [37]	74.4	2000	Jack‐knife	GLCM	MIAS
Fractal dimension by blanket [40]	75.74	2009	SVM	Fractal dimension by blanket	CHRU lille France
Lightweight CNN‐based U‐Net to refine the segmentation [51]	83.62	2025	Hybrid convolutional neural network (CNN)	Dice coefficient	Breast and thyroid data
A cancer diagnosis by CSVM [50]	84	2024	CSVM	Analysis of variance	SQUH
Convolutional neural networks using GPU [54]	84	2022	Neuronal convolutif hybride (CNN)	GPU	UFPR
Fractal dimension by variance [40]	85.5	2009	SVM	Fractal dimension by variance	CHRU lille France
Used grayscale reduction, by co‐occurrence matrix and SVM [46]	85.7	2013	SVM	Co‐occurrence matrix	MIAS
With the preprocessing of two techniques by BEMD [47]	86.66	2019	SVM	GLCM + GLCM preprocessing by BEMD	MGM Hospital
CNN‐CBR system for mammogram classification [52]	86.71	2023	Case‐based reasoning (CBR)	Textual CBR + pipeline preprocessing	CBIS‐DDSM
With preprocessing by BEMD [47]	88.88	2019	SVM	GLCM preprocessing by BEMD	MIAS
Convolutional neural networks in breast histopathology by CSVM [53]	89.52	2019	MIL	Informative features	BreaKHis
With preprocessing by BEMD [47]	90	2019	SVM	GLCM + GLCM preprocessing by BEMD	DDSM
Multifractal measurement by opt‐mBm [40]	91	2009	SVM	Hölder with opt‐mBm	CHRU lille France
With preprocessing by MBEMD [47]	92.59	2019	SVM	GLCM + preprocessing by MBEMD	MIAS
With preprocessing by MBEMD [47]	93.33	2019	SVM	GLCM + preprocessing by MBEMD	DDSM
Eltoukhy and Faye with KNN [43]	93.37	2013	KNN	Curvelet and wavelet	MIAS
Texture‐based features for mammogram classification using a decision tree [38]	93.6	2013	Decision tree	GLCM and GLRLM	Mammogram images
Texture features based on Gabor filters [35]	93.95	2016	SVM	Gabor filter	DDSM
Geometric and textural characteristics with SVM [36]	94	2014	SVM	Geometric characteristics	Mammogram images
Texture features combined by a Markov (MRF), a cancer diagnosis by CSVM [49]	94	2010	BPNN network	Markov (MRF) and fractal models	MIAS
Distribution of multifractal spectrum [41]	94.8	2023	SMFS	Multifractal spectrum	BUSI public
Digital mammograms using the fractal dimension measurements of BEMD [48]	95	2018	SVM	Fractal dimension preprocessing BEMD	DDSM
Multifractal measurement by opt‐mBm + variance [40]	95.45	2009	SVM	Hölder with opt‐mBm + variance	CHRU lille France
Classification of breast masses on mammograms using CVM [44]	95.89	2000	SVM	Energy laws + preprocessing by global thresholding	MIAS.
Contourlet‐based mammography mass classification using the SVM [39]	96.6	2010	SVM	Contour coefficients	MIAS
Eltoukhy and Faye with SVM [43]	96.66	2013	SVM	Curvelet and wavelet	MIAS
**Proposed method (MF-BIMFs) with SVM**	**97.32**	**—**	**SVM**	**Multifractal measurement + preprocessing by BEMD**	**RCRHKM**
MF‐RECO‐Img with SVM	73.09	—	SVM	Multifractal measurement + preprocessing by BEMD	RCRHKM
MF‐ORIG‐Img with SVM	63.6	—	SVM	Multifractal measurement	RCRHKM

*Note:* The bold data represents the highest value of the qualification rate obtained by our proposed method.

This combination improves classification performance, as the multifractal spectrum is used to extract relevant features from mammographic images. These features enable better abnormality detection and more accurate diagnoses. Thanks to the high visual similarity, new textural properties revealing changes in the breast were extracted from the spectrum. The results demonstrate the effectiveness of analyzing multifractal spectrum properties with BMED, enabling better characterization and assessment of breast cancer and, therefore, better differentiation between healthy and pathological cases, thanks to optimal use of decomposition information.

Our characterization approach, after applying the BEMD algorithm to obtain a multiresolution representation of the mammographic region, extracts multiscale multifractal spectrum measurements instead of using a single multifractal spectrum value extracted from the original image.

Our results are very satisfactory, considering the difficulty of image analysis and their high visual similarity. The features extracted from the multifractal spectral structure, especially the coefficients obtained with the BIMF_1_ mode decomposition, achieved the best accuracy scores (97.32%). The BIMF_1_ mode contains the image information and also reflects the high‐frequency components of the data. Compared to other modes, it can capture finer and more precise details. The experimental results show that feature extraction based on multifractal analysis, combined with BMED preprocessing, is a powerful and practical approach for the automatic classification of breast masses in mammograms. These features quantify the texture of an image and provide information on the spatial arrangement of pixels. They are important for image analysis and recognition. This system constitutes an accurate automatic diagnostic tool. This method can also detect breast cancer. They offer innovative solutions to improve feature extraction, classification accuracy, and selection of relevant data.

This system has demonstrated significant potential for cancer detection from digital mammograms. The proposed system achieves its primary objective: reducing computational and memory resource requirements for large training datasets and improving classification accuracy. By integrating these advanced techniques, this approach improves image clarity and helps radiologists make more informed and timely decisions.

### 6.5. Integration of the New System Into Diagnostic Workflows

According to Figure 18a, which presents the results obtained from the projections of the two healthy and cancerous populations for the proposed methods (MF‐BIMFs) at the BMIF_1_ level, we have seen that it seems quite effective in discriminating the pixel belonging to the tumor from those that do not belong to it, and also, we have obtained a higher classification rate of 97.32.

We can define a discrimination threshold which can help to detect pathological images defined by the following (Equation 39):

(39) Threshold=αmax+αmin2,

where *α*_min_ is the minimum value of the healthy multifractal spectrum at BIMF_1_ and *α*_max_ is the maximum value of the pathologies of the multifractal spectrum at BIMF_1_.

We have a malignant mammogram if one of the BIMF_1_ of the processed mammographic image has a multifractal spectrum *α* lower than this threshold.

This threshold can help radiologists assess breast images by providing a percentage of the risk of malignancy of a lesion. It can also reduce reading time and improve detection. Our study has the potential to make a significant contribution to the field of medical imaging and breast cancer diagnosis by providing a novel CAD system dedicated to breast mammographic images and AI techniques to manage decision‐making and to accurately classify mammographic images. This could ultimately lead to better early detection and treatment of breast cancer. The results obtained add learning technique through applications capable of creating rules (induction), which allow to deduce the appropriate action plan for a sequence of data points (incoming data) from the data points (outgoing data); the numbering of medical images allows to develop machine learning algorithms for their use and parameters; these algorithms can be used for the automatic processing of mammographic medical images through the use of natural language data and deep learning techniques, which can automatically observe breast cancers compared to radiologist cells.

The effectiveness of the proposed method is not limited to mammography and does not aim to replace radiologists, but by offering more accurate diagnoses, especially for dense breasts, which can be difficult to assess, it assists radiologists in evaluating images, providing a percentage of risk of malignancy of a lesion.

## 7. Conclusion and Outlook

This study presents a novel approach for breast cancer classification by analyzing mammographic images from the RCRHK dataset in Morocco. By integrating advanced image quality enhancement techniques with BEMD decomposition algorithms, multifractal parameter extraction was then performed for each BIMF. The feature classification results at the BIMF_1_ level have high accuracy compared to other modes. This is because higher spatial frequencies are correlated with lower modes that contain edge information. The proposed model demonstrates significant improvements in accuracy (97.32%) and diagnostic efficiency compared to existing state‐of‐the‐art approaches. This improvement process, combined with the integration of the feature extraction step with the BEMD preprocessing step, may prove essential in some applications to improve image quality, enrich the mathematical operations of the multifractal spectrum, and increase the performance of characterizing both healthy and cancerous populations. These results indicate that the proposed model has the potential to significantly improve breast cancer detection and classification in clinical settings. By providing rapid and accurate analysis of mammograms, this approach could promote earlier diagnosis and treatment of breast cancer, potentially improving patient outcomes. Establish a body of research to improve this process in the future, as well as suggest the use of alternative and more efficient preprocessing methods. Furthermore, we propose to incorporate more descriptors into the characterization step to improve the classification capability of breast masses.

## Conflicts of Interest

The authors declare no conflicts of interest.

## Funding

No funding was received for this manuscript.

## Data Availability

Data is available on request from the authors.
